# Healthy dietary indices and risk of depressive outcomes: a systematic review and meta-analysis of observational studies

**DOI:** 10.1038/s41380-018-0237-8

**Published:** 2018-09-26

**Authors:** Camille Lassale, G. David Batty, Amaria Baghdadli, Felice Jacka, Almudena Sánchez-Villegas, Mika Kivimäki, Tasnime Akbaraly

**Affiliations:** 1 Department of Epidemiology and Public Health, University College London, London, WC1E 6BT United Kingdom; 2 Department of Behavioural Science and Health, University College London, London, WC1E 6BT United Kingdom; 3 Department of Psychiatry & Autism Resources Centre, University Hospital of Montpellier, CHRU de Montpellier, F-34000 France; 4 INSERM, U1018, Centre for Research in Epidemiology and Population Health, Hôpital Paul Brousse, Villejuif, France; 5 Deakin University, Food & Mood Centre, IMPACT Strategic Research Centre, School of Medicine, Barwon Health, Geelong, Australia; 6 Nutrition Research Group, Research Institute of Biomedical and Health Sciences, University of Las Palmas de Gran Canaria, Las Palmas de Gran Canaria, Spain; 7 Ciber de Fisiopatología de la Obesidad y Nutrición (CIBER OBN), Instituto de Salud Carlos III, Madrid, Spain; 8 Clinicum, Faculty of Medicine, University of Helsinki, Helsinki, Finland; 9 MMDN, University of Montpellier, EPHE, INSERM, U1198, Montpellier, F-34095 France

**Keywords:** Depression, Physiology

## Abstract

With depression being the psychiatric disorder incurring the largest societal costs in developed countries, there is a need to gather evidence on the role of nutrition in depression, to help develop recommendations and guide future psychiatric health care. The aim of this systematic review was to synthesize the link between diet quality, measured using a range of predefined indices, and depressive outcomes. Medline, Embase and PsychInfo were searched up to 31^st^ May 2018 for studies that examined adherence to a healthy diet in relation to depressive symptoms or clinical depression. Where possible, estimates were pooled using random effect meta-analysis with stratification by observational study design and dietary score. A total of 20 longitudinal and 21 cross-sectional studies were included. These studies utilized an array of dietary measures, including: different measures of adherence to the Mediterranean diet, the Healthy Eating Index (HEI) and Alternative HEI (AHEI), the Dietary Approaches to Stop Hypertension, and the Dietary Inflammatory Index. The most compelling evidence was found for the Mediterranean diet and incident depression, with a combined relative risk estimate of highest vs. lowest adherence category from four longitudinal studies of 0.67 (95% CI 0.55–0.82). A lower Dietary Inflammatory Index was also associated with lower depression incidence in four longitudinal studies (relative risk 0.76; 95% CI: 0.63–0.92). There were fewer longitudinal studies using other indices, but they and cross-sectional evidence also suggest an inverse association between healthy diet and depression (e.g., relative risk 0.65; 95% CI 0.50–0.84 for HEI/AHEI). To conclude, adhering to a healthy diet, in particular a traditional Mediterranean diet, or avoiding a pro-inflammatory diet appears to confer some protection against depression in observational studies. This provides a reasonable evidence base to assess the role of dietary interventions to prevent depression. This systematic review was registered in the PROSPERO International Prospective Register of Systematic Reviews under the number CRD42017080579.

## Introduction

Depression, characterized by low mood, loss of interest or pleasure in life, and disturbed sleep or appetite, affects over 300 million people globally [1], which represents a global prevalence of 7% for women and 4% for men [2]. Depression is a leading cause of disease burden and a major contributor to global disability [3]. According to the World Health Organization, depressive and anxiety disorders cost the global economy $1 trillion in lost productivity each year [4].

Despite significant developments, conventional treatment is effective only in one in three cases of mood disorder [5]. Moreover, the condition is often recurrent, with relapse apparent in 50% of cases [6]. In this context, identifying modifiable risk factors to guide intervention strategies to prevent mood disorders and decrease their severity would appear to have value. A range of demographic, biological, genetic and behavioral determinants for depression have been proposed [7–10]. Of the latter, habitual diet has been increasingly examined as a potential independent predictor of disease risk (e.g., [11]) and the dietary intake of specific nutrients such as n-3 polyunsaturated fatty acids, B vitamins, zinc, and magnesium have been implicated in brain function [9, 12–15]. The neurological pathways potentially affecting depression risk that can be modulated by nutritional intake are related to inflammation, oxidative stress, neuroplasticity, mitochondrial function, and the gut microbiome [9].

The major challenge in understanding the role of diet in the etiology of chronic disease, including depression, is the characterization of this complex exposure. One approach is the identification of dietary patterns in the population under study by statistical modeling such as factor analysis (empirically-derived dietary patterns). These dietary patterns closely match the dietary habits of the studied population but do not necessarily reflect an optimal diet and are hardly replicable to other populations. In contrast to these a posteriori methods, a priori methods generate dietary indices based on existing knowledge of what constitutes a “healthy” diet (hypothesis-oriented dietary patterns). Based on a limited set of specific food groups, rather than specific nutrients, dietary indices reflecting adherence to an ideal diet can be very useful for clinicians to communicate with patients. Recent reviews have shown that healthy dietary patterns are associated with a decreased risk of depression or depressive disorders [16–20], but these are not universal findings [16]. Additionally, comparability of the studies is hampered by the variability in methodology and combination of estimates obtained using both hypothesis-oriented and data-driven dietary patterns. There is only one review of literature using a priori defined scores based on adherence to a traditional Mediterranean diet in relation to depression [21]. However, no formal comparison of the Mediterranean diet score exists with other widely used diet quality scores as, to the best of our knowledge, there is no exhaustive review of all a priori diet quality indices.

Accordingly, we provide a systematic review of studies assessing whether adherence to various dietary guidelines or traditional dietary patterns is associated with depressive symptoms and depression. For each dietary index we conducted a meta-analysis of results from observational studies. With the relationship likely to be bidirectional—mood can induce changes in eating behavior—only longitudinal studies can clarify the direction of the association. We therefore stratified our results according to study design.

## Methods

This systematic review was conducted following the Preferred Reporting Items for Systematic reviews and Meta-Analyses statement [22] and was registered in the PROSPERO International Prospective Register of Systematic Reviews (# CRD42017080579 at www.crd.york.ac.uk/PROSPERO).

### Search strategy

We used a four-pronged approach to identifying relevant publications. First, we searched Medline, Embase and PsychINFO via Ovid (http://www.ovid.com) for articles published since their inception (1946) to 31^st^ May 2018. The following keywords and index terms were used (“depression” or “depress* symptom*”) AND (“diet*”) AND (“index*” or “score*” or “pattern*” or “quality”). The search was limited to articles published in the English language and to full-text articles (conference abstracts were not considered). Second, we scrutinized the reference sections of the retrieved articles and systematic reviews. Third, we contacted experts in the field. Fourth, we searched our own files. The search was conducted in parallel by two authors (TA and CL) with any disagreements resolved by a third (AB).

### Study selection

To be included in this systematic review, articles had to meet the following criteria: (1) Exposure: comprehensive dietary assessment (food frequency questionnaire, 24-h diet recall, food record, diet history) and use of an a priori dietary score or index; (2) Outcome: *clinical depression* assessed by the study staff, medical records or self-reported clinician-diagnosed (e.g., “Have you ever been diagnosed with depression by a medical doctor?”), *depressive symptoms* assessed by validated scales/questionnaires (such as the Center for Epidemiological Studies Depression scale, CES-D), and defined according to validated cutoffs of these scales (i.e., cutoff of 16 for the 20-item CES-D scale) and *the use of anti-depressive drugs* considered only when it was combined to clinical depression or depressive symptoms assessment; (3) Design: observational study (cross-sectional, cohort, case-control); (4) Population: general free-living populations without any age limit that includes outpatients (individuals not hospitalized for physical or mental health reasons) and non-institutionalized individuals. If the study had any of the following characteristics, it was excluded: (1) Exposure: no measure of whole diet (e.g., dietary screeners or individual questions) or use of a posteriori dietary patterns; (2) Outcome: bipolar disorders, overall mood states, psychosocial stressors or perceived stress; (3) Design: intervention studies; (4) Population: pregnant or lactating women, inpatient/hospitalized populations.

### Data extraction

After study selection, the following information was extracted from each retrieved article: first author’s surname, journal, year published, geographical location, study design, follow-up time (if applicable), sex and mean age, sample size, number of cases, dietary assessment tool, dietary score used (range and mean score), assessment of depression, depressive symptoms scale and threshold used (if symptoms), modeling strategy, confounders used, main findings including odds ratios or hazard/risk ratios and their standard errors/confidence intervals. When a study provided several estimates, we chose to use those from the most complex model (that is, the one including the largest number of confounders).

### Quality and risk of bias assessment

We adapted the Newcastle-Ottawa checklist [23] to assess whether cohorts were representative of the wider population (as opposed to a selected occupational group, for instance), if diet was ascertained by means of a validated dietary assessment tool (e.g., FFQ), if the dietary score used was validated, whether follow-up was sufficient to preclude reverse causation (≥5 years), and if appropriate statistical adjustment was made (age, sex, smoking, physical activity, body mass index, total energy intake). If at least four of the five of the above criteria was met, the study was considered of high quality and to be at low risk of bias.

### Statistical analysis

For each dietary score (exposure variable), we conducted separate meta-analyses dependent on study design (cross-sectional vs. longitudinal). We combined the results of the studies that presented analyses with the dietary score as a categorical variable, computing forest plots and combined odds, hazard or risk ratios for depression for the healthiest compared to the least healthy category. When the dietary score was analyzed as a continuous variable (four studies [24–27]), the estimates were not included in the calculation of the combined results. Estimates (beta and standard error) from studies that used depressive symptoms (outcome) as a continuous variable were converted into log odds ratios by multiplying by a factor 1.81 and then exponentiated [28]. We used random-effect meta-analysis models to account for potential heterogeneity and assessed heterogeneity by the *I*^2^ statistic [29]. Potential for publication bias was examined using contour-enhanced funnel plots where asymmetry and absence of studies in areas of non-significance suggest the presence of reporting bias [30]. We also calculated the Egger and Begg test for small study effect. Stata version 14 (StataCorp, TX, USA) was used for the statistical analyses.

### Code availability

The Stata commands metan, confunnel and metabias were used. All codes used to generate the meta-analysis results can be obtained from the authors upon request.

### Sensitivity analyses

As results are stratified by dietary index and by study design, further stratification can lead to only one association estimate in some strata. Therefore, we only present the sensitivity analyses results in the subgroups that contain the majority of studies. To test the impact of outcome definition (clinical depression vs. depressive symptoms), of age of the participants, of geographical region (high vs. low-middle income countries) and of study quality, we performed sensitivity analyses by variously excluding: the studies using clinical depression as they were a minority; studies on adolescents; studies performed in low-middle income countries; and studies of low quality. Finally, a study using the Mediterranean diet was identified that used psychological distress as a marker of depression [31], so we also performed a sensitivity analysis by excluding this study for a more strict assessment of depression.

## Results

In Supplemental Fig. 1 we depict the process of study selection. The search yielded 3272 records (after exclusion of duplicates), of which 3058 were excluded after title and abstract screening. Out of the 214 full-text articles assessed for eligibility, we retained 51 that we scrutinized for methodological quality. The articles excluded are listed by reason of exclusion in Supplemental material. A total of ten articles were dropped after methodological quality check, comprising the presentation of only unadjusted comparisons between groups and no further statistical modeling in six studies [32–37], or no measure of whole diet in a further four [38–41]. This yielded a total 41 articles for this systematic review: 20 longitudinal and 21 cross-sectional studies.

The majority of studies were on generally healthy participants. Two studies involved participants at high risk of knee osteoarthritis [42, 43] and one included participants with a history of myocardial infarction [44]. Ten analyses used a Mediterranean diet score [25–27, 31, 43, 45–49], seven the Healthy Eating Index (HEI) or the Alternative Healthy Eating Index (AHEI) [48, 50–55], four a Dietary Approaches to Stop Hypertension (DASH) diet score [56–59], nine the Dietary Inflammatory Index (DII) [42, 60–67], and 15 a variety of scores such as adherence to national dietary guidelines or general “diet quality” [44, 46, 51, 68–79]. The components included in each of the main diet scores (Mediterranean, HEI, AHEI, DASH) are summarized in Supplemental Table 1. One study simultaneously captured three scores, a Mediterranean diet score, the HEI, and a pro-vegetarian dietary pattern [48], another compared the Mediterranean diet score with the Australian Recommended Food Score [46], and a last one compared the AHEI with three other scores [51].

We graded 32 analyses as being of high quality (scoring four or five) [25–27, 31, 42, 45, 46, 48, 49, 51, 55–58, 60–67, 69, 71–75, 77, 79], whereas 12 studies had a low quality score of three or less [43, 44, 47, 48, 50, 52–54, 59, 70, 76, 78], of which the majority (nine) were cross-sectional studies.

### Mediterranean diet

Adherence to a traditional Mediterranean diet was measured by four different indices: the original Mediterranean Diet Score (MDS) [25, 31, 46, 48] developed by Trichopoulou and colleagues [80], the relative Mediterranean diet score (rMED) [45], the alternative Mediterranean diet score (aMED) [26, 27, 43] or the Mediterranean Style Dietary Pattern Score (MSDPS) [49]. The MDS and rMED include nine items: five beneficial (fruit, vegetable, legumes, cereals, fish), two considered detrimental (meat, dairy), one component on fat (mono-unsaturated fatty acids/saturated fatty acids [MDS] or olive oil intake [rMED]) and one component on moderate alcohol intake. The MDS ranges from zero to nine points: one point is allocated if the intake is above the median, zero if below (or inversely for detrimental items). The rMED is based on tertiles as cutoffs, therefore ranges 0–18. The aMED scores from zero (lower adherence) to five (better adherence) on 11 components (same as MDS, adding poultry [detrimental] and potatoes [beneficial]), so the total score ranges 0–55. The MSDPS comprises 13 components (same as aMED, adding sweets and eggs), each scored continuously from 0 to 10 and the total score is standardized, ranging 0–100.

The study characteristics are given in Table 1. There were two reports from the same study (the Spanish Seguimiento Universidad de Navarra, SUN [47, 48]), so we included the one with the longest follow-up (8.5 years) [48]. In total, we considered data from six cohort studies comprising samples drawn from France [45], Australia [31, 46], Spain [48], the UK [25] and the US [27] (average 9.1 years of follow-up). There were three cross-sectional studies (the US [43], Greece [26] and Iran [49]).

**Table 1:** Characteristics of observational studies that examined the associations between healthy dietary indices and depressive outcomes

Author, year	Country	Design follow-up	Population, age	*N*, *n* cases	Dietary assessment	Dietary score	Depression assessment	Model	Adjustment	OR, HR or RR, or *β* coefficients
Mediterranean diet										
Adjibade 2017 [45]	France	Cohort 12.6 years	*Adults* SUVIMAX, 1994–2007, Men 52.1 years, women 47.6 years	Men 2031, 69 cases; women 1492, 103 cases	~10, 24-hDR over 2 years	rMED	Follow-up: Q. Men CES-D-20 ≥ 17; women CES-D-20 ≥ 23. Baseline: Q, A. Exclusion depressive symptoms (same CES-D cutoffs) or antidepressant use.	Logistic regression	Age, sex, intervention group, education, marital status, socio-professional status, energy intake, number of 24-h dietary records, interval between CES-D measurements, smoking status, physical activity, BMI	Men OR T3 vs. T1 0.58; 95% CI 0.29, 1.13, continuous OR 0.91; 95% CI 0.83, 0.99. Women OR T3 vs. T1 0.95; 95% CI 0.57, 1.59, continuous OR 0.99; 95% CI 0.91, 1.06
Hodge 2013 [31]	Australia	Cohort 11 years	*Adults* Melbourne Collaborative cohort study 1990–2007, 50–69 years	8660, 731 cases	121-item FFQ	MDS with olive oil	Follow-up: Q. K10 ≥ 20. Baseline: A. Exclusion antidepressant or anxiolytics use.	Logistic regression	Physical activity, education, smoking, history of arthritis, asthma, kidney, energy intake, SES	OR score 7–9 vs. 0–3: 0.72; 95% CI 0.54, 0.95
Sanchez Villegas 2009 [47]	Spain	Cohort 4.4 years	*Adults* SUN, 37.5 years	10,094, 480 cases	136-item FFQ	MDS	Follow-up: C, A. Self-reported doctor diagnosis or habitual use of antidepressant. Baseline: C, A. Exclusion antidepressant use or previous clinical diagnosis	Cox proportional hazards model	Age, sex, BMI, smoking, physical activity, vitamin supplements, energy intake, chronic disease at baseline	OR score 6–9 vs. 0–2: 0.58; 95% CI 0.44, 0.77
Sanchez Villegas 2015 [48]	Spain	Cohort 8.5 years	*Adults* SUN, 37.5 years	15,093, 1051 cases	As above	As above	As above	As above	As above	OR score 6–9 vs. 0–2: 0.70; 95% CI 0.58, 0.85
Lai 2016 [46]	Australia	Cohort 12 years	*Adults* ALSWH, 50–54 years	9280	DQES	MDS	Follow-up and baseline: Q. CES-D-10 continuous Adjustment for baseline depression (no exclusion)	Linear mixed model with time-varying covariates	Area of residence, marital status, income, education, physical activity, smoking, baseline self-reported physician depression diagnosis, antidepressant use	Q5 vs. Q1, *β* = −0.48; 95% CI −0.74, −0.21
Skarupski 2013 [27]	US	Cohort 7.2 years	*Adults* CHAP Chicago, 73.5 years	3502	139-item FFQ	aMED	Follow-up: Q. CES-D-10 continuous. Baseline: Q. Exclusion of CES-D-10 ≥ 4	Generalized estimating equations	Age, sex, race, education, income, widowhood, energy intake, BMI	Slope over time is positive in T1 but negative in T3, slope difference between T3 and T1 *β* = −0.03, SE = 0.01, *p* < 0.001
Winpenny 2018 [25]	UK	Cohort 3 years	*Adolescents* ROOTS, 14 years	603	4 day diet diary	MDS	Follow-up and baseline: Q. MFQ-33, continuous. Adjustment for baseline score (no exclusion)	Linear regression	Sex, SES, smoking, alcohol, physical activity, sleep, friendship quality, self-esteem, family functioning, medication use, % body fat, baseline MFQ score	Beta 1 SD MDS 0.35; 95% CI −0.04, 0.74
Veronese 2016 [43]	US	Cross-sectional	*Adults* Osteoarthritis initiative, 61.3 years. *Patients at high-risk of osteoarthritis*	4470	70-item FFQ	aMED	Q. CES-D-20 ≥ 16	Logistic regression	Age, sex, race, BMI, education, smoking, annual income, Charlson comorbidity index, analgesic drugs use, total energy intake	OR Q4–5 vs. Q1–3 0.82; 95% CI 0.65, 1.04
Mamplekou 2010 [26]	Greece	Cross-sectional	*Adults* MEDIS, 74 years	1190, 246 cases	FFQ	aMED	Q. GDS-15 > 10	Logistic regression	Age, sex, education, BMI, physical activity, hypertension, diabetes, hypercholesterolemia	OR 1 unit increase 1.03; 95% CI 0.98, 1.09
Tehrani 2018 [49]	Iran	Cross-sectional	*Adolescents* Tehran, 16.2 years	263	168-item FFQ	MSDPS	Q. DASS-21 ≥ 10 on the depression subscale	Logistic regression	Age, BMI, energy intake, physical activity, ethnicity, parents’ education level and total family income	OR Q5 vs. Q1 0.41; 95% CI 0.17, 0.97
Healthy Eating Index HEI/Alternative Eating Index AHEI										
Adjibade 2018 [51]	France	Cohort 5.9 years	*Adults* NutriNet-Santé, 2009–2018, 18–86 years Men 53.0 years, women 45.5 years	26,225, 2166 cases	~8, 24-hDR over the first 2 years	AHEI-2010	Follow-up: Q. Men CES-D-20 ≥ 17; women CES-D-20 ≥ 23. Baseline: Q, A. Exclusion depressive symptoms (same CES-D cutoffs) or antidepressant use.	Cox proportional hazards model	Age, sex, marital status, educational level, occupational categories, household income, residential area, energy intake without alcohol, number of 24hs and inclusion month, smoking, physical activity, BMI, health events during follow-up	HR T3 vs. T1 0.96; 95% CI 0.86, 1.07 .HR per 1 SD increase 0.98; 95% CI 0.94, 1.03
Akbaraly 2013 [55]	UK	Cohort 5 years	*Adults* Whitehall II 1991–2009, 61 years	Men 3155, 164 cases; women 1060, 96 cases	127-item FFQ	AHEI	Follow-up: Q, A. Recurrent (at both phase 7 and 9) depressive symptoms CES-D-20 ≥ 16 or antidepressant use. Baseline: A. Exclusion antidepressant use (phase 3 or 5)	Logistic regression	Age, sex, ethnicity, energy intake, SES, retirement, living alone, smoking, physical activity, CAD, diabetes, hypertension, HDL cholesterol, lipid-lowering drugs, central obesity, cognitive impairment	OR T3 vs. T1 Men 0.95; 95% CI 0.64, 1.42. Women 0.36; 95% CI 0.20, 0.64
Sanchez Villegas 2015 [48]	Spain	Cohort 8.5 years	*Adults* SUN, 37.5 years	15,093, 1051 cases	136-item FFQ	AHEI-2010	Follow-up: C, A. Self-reported doctor diagnosis or habitual use of antidepressant. Baseline: C, A. Exclusion antidepressant use or previous clinical diagnosis	Cox proportional hazards model	Age, sex, BMI, smoking, physical activity, vitamin supplements, energy intake, chronic disease at baseline	HR Q5 vs. Q1 0.72; 95% CI 0.59, 0.88
Loprinzi 2014 [52]	US	Cross-sectional	*Adults* NHANES 2005–2006, 20–85 years	2574, 118 cases	Two 24-hDR	HEI 2005	Q. PHQ-9 ≥ 10	Logistic regression	Age, gender, ethnicity, BMI, PIR, presence of comorbidities	OR > 60 percentile vs. below 0.51; 95% CI 0.27, 0.93
Rahmani 2017 [53]	Iran	Cross-sectional	*Adults* Iranian soldiers, 24 years	246, 39 cases	FFQ	AHEI-2010	Q. DASS-21 > 21 on the depression subscale (severe depression)	Logistic regression	Age, energy intake, BMI, physical activity, education, marital status, smoking, SES, family size	OR Q4 vs. Q1 0.12; 95% CI 0.02, 0.58
Saneei 2016 [54]	Iran	Cross-sectional	*Adults* Iranian adults, 36.3 years	Men 1403, 321 cases; women 1960, 688 cases	106-item FFQ	AHEI-2010	Q. Iranian HADS-21 ≥ 8	Logistic regression	Age, sex, energy intake, BMI, physical activity, smoking, marital status, educational level, family size, house possession, self-reported diabetes, current use of antipsychotic medications, dietary supplements	OR Q4 vs. Q1 Men 0.70; 95% CI 0.44, 1.11. Women 0.51; 95% CI 0.36, 0.71
Beydoun 2010 [50]	US	Cross-sectional	*Adults* HANDLS, 47.9 years	734, 21.2% men and 32.1% women	Two, 24-hDR	HEI-2005	Q. CES-D-20 ≥ 16	Linear regression	Age, ethnicity, marital status, education, poverty status, smoking status, illicit drug use, and BMI	Men *β* = −0.045 SE = 0.024 *p* = 0.06. Women *β* = −0.083, SE = 0.023, *p* < 0.001
Dietary approaches to stop hypertension DASH										
Perez Cornago 2017 [58]	Spain	Cohort 8 year	*Adults* SUN, 37.5 years	14,051, 113 cases	136-item FFQ	Dixon Mellen Fung Gunther	Follow-up: C, A. Self-reported doctor diagnosis or habitual use of antidepressant. Baseline: C, A. Exclusion antidepressant use or previous clinical diagnosis	Cox proportional hazards model	Sex, smoking, physical activity, energy intake, living alone, unemployment, marital status, baseline hypertension, weight change, personality traits	HR Dixon 3–9 vs.. ≤ 2, 1.47; 95% CI 0.95, 2.3 HR Mellen Q2–Q5 vs. Q1, 0.68; 0.45, 1.04 HR Fung Q2–Q5 vs. Q1, 0.63; 95% CI 0.41, 0.95 HR Gunther Q2–Q5 vs. Q1, 1.01; 95% CI 0.63, 162
Meegan 2017 [57]	Ireland	Cross-sectional	*Adults* Cork&Kerry Diabetes and Heart Disease Study, 59.8 years	2040, 302 cases	127-item EPIC FFQ	Fung	Q. CES-D-20 ≥ 16	Logistic regression	Age, sex, BMI, smoking, physical activity, alcohol, antidepressant use, history of depression	OR > median vs. below 1.06; 95% CI 0.69, 1.63
Valipour 2017 [59]	Iran	Cross-sectional	*Adults* SEPAHAN, 36 years	1712 men; 2134 women 1108 total cases	106-item FFQ	Fung modified (deciles, different items)	Q. HADS-D-21 ≥ 8	Logistic regression	Age, sex, energy intake, marital status, socioeconomic status, smoking, physical activity, chronic disease, antidepressant use, supplement use, pregnant or lactating, frequent spice consumers, BMI	OR ≥ 51 vs. ≤ 40: men 1.08; 95% CI 0.73, 1.6, women 0.96; 95% CI 0.72, 1.28
Khayyatzadeh 2017 [56]	Iran	Cross-sectional	*Adolescents* Iranian girls, 12–18 years	535, 172 cases	168-item FFQ	Fung	Q. Persian version of BDI-21 > 16	Logistic regression	Age, energy intake, mother job status, passive smoker, menstruation, parent death, parent divorce, physical activity, BMI, SES, education	OR Q4 vs. Q1 0.47; 95% CI 0.23, 0.92
Dietary Inflammatory Index DII										
Akbaraly 2016 [61]	UK	Cohort 5 years	*Adults* Whitehall II 2002–2009, 61 years	Men 3178, 166 cases; Women 1068, 99 cases	127-item FFQ	DII	Follow-up: Q, A. Recurrent (phase 7 and 9) depressive symptoms CES-D-20 ≥ 16 or antidepressant use. Baseline: A. Exclusion antidepressant use (phase 3 or 5)	Logistic regression	Age, sex, ethnicity, marital status, occupation, smoking, alcohol, energy intake, physical activity, CVD risk factors	OR T1 vs. T3 men 0.96; 95% CI 0.60, 1.54. Women 0.35; 95% CI 0.18, 0.68
Adjibade 2017 [60]	France	Cohort 12.6 years	*Adults* SUVIMAX, 1994–2007, Men 52.1, Women 47.6	Men 2031, 69 cases; Women 1492, 103 cases	~10 24-hDR over 2 years	DII	Follow-up: Q. Men CES-D-20 ≥ 17; women CES-D-20 ≥ 23. Baseline: Q, A. Exclusion depressive symptoms (same CES-D cutoffs) or antidepressant use.	Logistic regression	Age, sex, intervention group, education, marital status, socio-professional status, energy intake, number of 24-h DR, interval between the two CES-D measurements, smoking, physical activity, BMI	OR Q1 vs. Q4 men 0.43; 95% CI 0.19, 0.99. Women 1.39; 0.75, 2.56
Sanchez Villegas 2015 [67]	Spain	Cohort 8.5 years	*Adults* SUN, 37 years	15,093, 1051 cases	136-item FFQ	DII	Follow-up: C, A. Self-reported doctor diagnosis or habitual use of antidepressant. Baseline: C, A. Exclusion antidepressant use or previous clinical diagnosis	Cox proportional hazards model	Age, sex, energy intake, prevalence of disease, BMI, smoking, physical activity, vitamin supplement, CVD, diabetes, hypertension, dyslipidemia at baseline	HR Q1 vs. Q5 0.68; 95% CI 0.54, 0.85
Shivappa 2016 [65]	Australia	Cohort 9 years	*Adults* ALSWH 2001–2013, 52 years	6438, 1156 cases	101-item FFQ	DII	Follow-up: Q. CES-D-10 ≥ 10 Baseline: Q. Exclusion history depressive symptoms CES-D-10 ≥ 10 survey 1 to 3.	Relative risk (log-binomial or Poisson)	Energy intake, education, marital status, menopause status, night sweats and major personal illness or injury, smoking, physical activity, BMI, depression diagnosis or treatment	RR Q1 vs. Q4 0.81; 95% CI 0.69, 0.96
Shivappa 2018 [42]	US	Cohort 8 years	*Adults* Osteoarthritis initiative, 61.4 years *Patients at high-risk of osteoarthritis*	3608, 837 cases	70-item FFQ	DII	Follow-up: Q. CES-D-20 > 16. Baseline: Q. Exclusion prevalent depressive symptoms CES-D-20 > 16	Cox proportional hazards model	Age, sex, race, BMI, education, smoking, income, physical activity, Charlson co-morbidity index, CES-D at baseline, statin use, NSAIDS or cortisone use	HR Q1 vs. Q4 0.81; 95% CI 0.65, 0.99
Wirth 2017 [66]	US	Cross-sectional	*Adults* NHANES III, 2005–2012, 46.9 years	Men 9322, 595 cases; women 9553, 1053 cases	Two 24-hDR	DII	Q. PHQ-9 ≥ 10	Logistic regression	Race, education, marital status, perceived health, current infection status, smoking family member, smoking status, past cancer, diagnosis, arthritis, age, average nightly sleep duration	OR Q1 vs. Q4 Men 0.92; 95% CI 0.61, 1.37. Women 0.77; 95% CI 0.60, 1.00
Bergman 2017 [62]	US	Cross-sectional	*Adults* NHANES III, 2007–2012, 20–80 years	11,592, 939 cases	Two 24-hDR	DII	Q. PHQ-9 ≥ 10	Logistic regression	Age, sex, ethnicity, poverty income ratio, employment, health insurance, education, marital status, BMI, smoking, physical activity, sedentary time, vitamin supplements use, energy intake, menopause, comorbidity (hypertension, hyperlipidemia, diabetes, CVD, respiratory illness, cancer)	OR Q1 vs. Q5 0.44; 95% CI 0.31, 0.63
Philipps 2017 [63]	Ireland	Cross-sectional	*Adults* Cork&Kerry Diabetes and Heart Disease Study, 50–69 years	1992	127-item EPIC FFQ	DII	Q. CES-D-20 ≥ 16	Logistic regression	Age, sex, BMI, physical activity, smoking, alcohol consumption, antidepressant use and history of depression	OR T1 vs. T3 men 1.28; 95% CI 0.61, 2.78. Women 0.45; 95% CI 0.23, 0.87
Shivappa 2017 [64]	Iran	Cross-sectional	*Adolescents* Iranian adolescent girls, 15–18 years	299, 84 cases	168-item FFQ	DII	Q. DASS-21 > 9	Logistic regression	Age, total energy intake, physical activity, marital status, income, smoking, BMI, chronic disease	OR T1 vs. T3 0.29; 95% CI 0.11, 0.75
Other diet quality indices										
Collin 2016 [69]	France	Cohort 13 years	*Adults* SUVIMAX, 49.5 years	3328, 340 cases	~10, 24-hDR over 2 years	mPNNS-GS French guidelines	Outcome: Q. chronic depressive symptoms defined as CES-D-20 ≥ 16 at baseline and follow up. Baseline: A. Exclusion of antidepressant use.	Logistic regression	Age, sex, energy intake, education, marital status, tobacco, supplementation group, number of 24 h DR, baseline BMI and physical activity	OR Q4 vs. Q1 0.51; 95% CI 0.35, 0.73
Adjibade 2018 [51]	France	Cohort 5.9 years	*Adults* NutriNet-Santé, 2009–2018, 18–86 years. Men 53.0 years, women 45.5 years	26,225, 2166 cases	~8, 24-hDR over the first 2 years	mPNNS-GS French guidelines	Follow-up: Q. Men CES-D-20 ≥ 17; women CES-D-20 ≥ 23 Baseline: Q, A. Exclusion depressive symptoms (same CES-D cutoffs) or antidepressant use.	Cox proportional hazards model	Age, sex, marital status, educational level, occupational categories, household income, residential area, energy intake without alcohol, number of 24hs and inclusion month, smoking, physical activity, BMI, health events during follow-up	HR T3 vs. T1 0.80; 95% CI 0.72, 0.90. HR per 1 SD increase 0.92; 95% CI 0.87, 0.96
						PANDiet				HR T3 vs. T1 0.88; 95% CI 0.79, 0.98. HR per 1 SD increase 0.95; 95% CI 0.91, 0.99
						DQI-I				HR T3 vs. T1 0.79; 95% CI 0.70, 0.88. HR per 1 SD increase 0.91; 95% CI 0.87, 0.95
Lai 2017 [77]	Australia	Cohort 9 years	*Adults* ALSWH, Women 45–50 years	7877, 2841 cases	DQES	ARFS	Follow-up: Q. CES-D-10 ≥ 10. Baseline: A. Exclusion self-report depression	Logistic regression	Area of residence, marital status, income, education, smoking, physical activity, anxiety/nervous disorder	OR T3 vs. T1 0.94; 95% CI 0.83, 1.00
Lai 2016 [46]	Australia	Cohort 12 years	*Adults* ALSWH, Women 45–50 years	11,046	DQES	ARFS	Follow-up and baseline: Q. CES-D-10 continuous Adjustment for baseline depression (no exclusion)	Linear mixed model	Area of residence, marital status, income, education, smoking, physical activity, self-reported physician diagnosis and use of antidepressants	Q5 vs. Q1 *β* = −0.23; 95% CI −0.47, 0.01
Espana Romero 2013 [70]	US	Cohort 6.1 years	*Adults* Aerobics Center Longitudinal Study, 47 years	5110, 641 cases	3 day food record	AHA diet goals. 4 items: F&V, fish, sodium, wholegrain	Follow-up: Q. CES-D-30 ≥ 8. Baseline: C. Exclusion previous mental disorder	Logistic regression	Age, sex, baseline year, heavy alcohol intake, other ideal components: smoking, BMI, physical activity, total cholesterol, blood pressure, fasting plasma glucose	OR ideal (3–4) vs. poor (0–1) 0.58; 95% CI 0.37, 0.92
Gall 2016 [71]	Australia	Cohort 5 years	*Adults* Childhood Determinants of Adult Health (CDAH), 31.7 years	1233, 203 cases	127-item FFQ	Australian Dietary Guideline Index	C. Composite International Diagnostic Interview diagnosis of major depression. Outcome: first episode vs. no new episode (may include history of mood disorder before baseline)	Log multinomial regression	Age, sex, education, physical health related quality of life, history of CVD or diabetes, oral contraceptive use, area-level SES, social support, parental status	RR Q4 vs. Q1–Q3: 0.71; 95% CI 0.39, 1.3
Sanchez Villegas 2015 [48]	Spain	Cohort 8.5 years	*Adults* SUN, 37.5 years	15,093, 1051 cases	136-item FFQ	Pro-vegetarian food pattern	Follow-up: C, A. Self-reported doctor diagnosis or habitual use of antidepressant. Baseline: C, A. Exclusion antidepressant use or previous clinical diagnosis	Cox proportional hazards model	Age, sex, BMI, smoking, physical activity, vitamin supplements, energy intake, chronic disease at baseline	HR Q5 vs. Q1 0.78; 95% CI 0.64, 0.93
Voortman 2017 [79]	Netherlands	Cohort 13.5 years	*Adults* Rotterdam Study, 64.1 years	6217, 1686 cases	389-item FFQ	Dutch Dietary Guidelines 2015	Follow-up: C, A. Self-reported history of depression, psychiatric examination (CES-D + semi-structured clinical interview), medical records, antidepressant. Baseline: C. Exclusion prevalent disease	Cox proportional hazards model	Cohort, age, sex, education, employment, smoking, physical activity and energy intake	HR Q5 vs. Q1 0.89; 95% CI 0.76, 1.04
Jacka 2010 [75]	Australia	Cross-sectional	*Adults* Geelong Osteoporosis Australia, Women 20–94 years	1046, 60 cases	74-item FFQ	ARFS	C. Structured clinical interview for DSM-IV-TR	Logistic regression	Age, socioeconomic status, education, physical activity, alcohol, smoking, energy intake	OR *z*-score, 0.85; 95% CI 0.62, 1.13
							Q. GHQ-12 continuous	Linear regression	As above	*β* = −0.08; 95% CI −0.14, -0.01
Jacka 2011 [74]	Norway	Cross-sectional	*Adults* Hordaland Health study 1997, 46–74 years	Men 2477, 230 cases. Women 3254, 281	169-item FFQ	DQS 6 items	Q. HADS-D-7 ≥ 8	Logistic regression	Age, income, education, physical activity, smoking, alcohol, energy intake	OR *z*-score Men 0.83; 95% CI 0.70, 0.99. Women 0.71; 95% CI 0.59, 0.84
Sakai 2017 [78]	Japan	Cross-sectional	Three-generation Study of Women on Diets and Health, *Adolescenst* 18 years. *Adults* 47.9 years	Adolescent 3963, 871 cases. Adults 3833, 643 cases	DHQ	Japanese DQS 7 items	Q. CES-D-20 ≥ 23	Logistic regression	BMI, smoking, medication use, self-reported stress, dietary reporting status, physical activity, energy intake	OR Q5 vs. Q1 Adolescents 0.67; 95% CI 0.49, 0.92. Adults 0.55; 95%CI 0.4, 0.75
Gomes 2017 [72]	Brazil	Cross-sectional	*Adults* Pelotas, 60 years+	Men 508, 50 cases. Women 870, 161 cases	FFQ	EDQ-I	Q. Brazilian GDS-10 ≥ 5	Logistic regression	Age, marital status, education, economic class, leisure time physical activity, current smoking, alcohol intake	OR T1 vs. T3 Men 3.78; 95% CI 1.35, 10.57. Women 2.13; 95%CI 1.35, 3.33
Huddy 2016 [73]	Australia	Cross-sectional	*Adults* Melbourne InFANT Extend Program, Women 19–45 years	437, 151 cases	137-item FFQ	Australian Dietary Guideline Index	Q. CES-D-10 ≥ 10	Linear regression	Age, education, smoking, physical activity, television viewing, sleep quality, BMI	1 point increase *β* = −0.034; 95%CI −0.056, −0.012
Kronish 2012 [76]	US	Cross-sectional	*Adults* REGARDS 2003–2007, 65 years	20,093, 1959 cases	109-item FFQ	5 items: fish, F&V, sodium, sugar, fiber/carb	Q. CES-D-4 ≥ 4	Poisson regression	Age, race, sex, region of residence, income, education	Prevalence ratio < 2 vs. ≥ 2 1.08; 95% CI 1.06, 1.10
Rius-Ottenheim 2017 [44]	Netherlands	Cross-sectional	*Adults* Alpha Omega, 72.2 years *Patients with a history of myocardial infarction*	2171	203-item FFQ	DHNaFS DUNaFS	Q. GDS-15 continuous	Linear regression	Age, sex, education, marital status, physical activity, BMI, high alcohol use, smoking, antidepressants use, family history of depression, self-rated health, chronic disease, treatment group	continuous, *β* = −0.108 *p* < 0.001; continuous, *β* = −0.002 *p* = 0.93

Country: *UK* United Kingdom, *US* United States of America

Study: *ALSWH* Australian Longitudinal Study on Women's Health, *HANDLS* Healthy Aging in Neighborhoods of Diversity across the Life Span; *InFANT* Infant Feeding, Activity, and Nutrition Trial, *MEDIS* MEDiterranean ISlands study, *NHANES* National Health and Nutrition Examination Survey, *REGARDS* Reasons for Geographic and Racial Differences in Stroke, *ROOTS* adolescents from Cambridgeshire and Suffolk recruited through secondary schools, *SEPAHAN* Studying the Epidemiology of Psycho-Alimentary Health and Nutrition, *SUN* Seguimiento Universidad de Navarra, *SUVIMAX* Supplementation en Vitamines et Mineraux study

Dietary instrument: *24h* DR 24-hour dietary recall, *DHQ* diet history questionnaire, *DQES* dietary questionnaire for epidemiological studies, *FFQ* food frequency questionnaire

Dietary score: *MDS* Mediterranean Diet Score, *rMED* relative Mediterranean Diet Score*, aMED* alternative Mediterranean Diet Score, *AHEI* Alternative Healthy Eating Index, *HEI* Healthy Eating Index, *DASH* Dietary Approaches to Stop Hypertension, *DII*, Dietary Inflammatory Index, *mPNNS-GS* modified score to the French dietary guidelines (PNNS-Guideline Score), *AHA*, American Heart; Association, *ARFS* Australian Recommended Food Score, *DGI*, Dietary Guidelines Index, *DQI-I*, Diet Quality Index International, *DQS* diet quality score, *DHNaFS* Dutch Healthy Nutrient and Food Score, *DUNaFS* Dutch Undesirable Nutrient and Food Score, *EDQ-I* Elderly Dietary Quality Index, *PANDiet* Diet Quality Index based on the probability of adequate nutrient intake

Depression assessment: *Q* questionnaire, *C* clinical, *A* antidepressant use, *BDI* Beck Depression Inventory, *CES-D* Center for Epidemiological Studies Depression scale (followed by number of items in the scale), *DASS* Depression Anxiety and Stress Scale, *DSM-IV-TR* Diagnostic and Statistical Manual of Mental Disorders, *GDS* Geriatric Depression Scale, *GHQ-12* General Health Questionnaire 12 items, *HADS(-D)* Hospital Anxiety and Depression Scale (Depression subscale), *K10* Kessler Psychological Distress Scale, *MFQ* Moods and Feelings Questionnaire (range 0–66), *PHQ-9* Patient Health Questionnaire 9 item depression module

Adjustment: *BMI* body mass index, *CAD* coronary artery disease, *CVD* cardiovascular disease, *HDL* high-density lipoprotein, *PIR* poverty-to-income ratio, *SES* socioeconomic status

Measure of association: *OR* odds ratio, *HR* hazard ratio, *RR* risk ratio, *95% CI* 95% confidence interval, *T* tertile, *Q4* quartile, *Q5* quintile

**Fig. 1:**
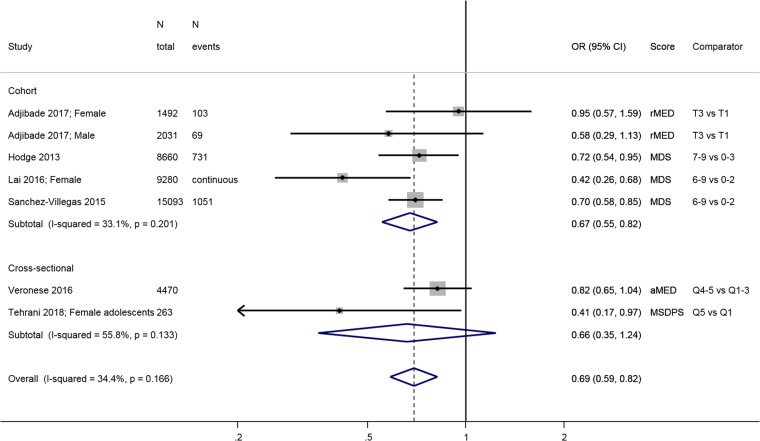
Meta-analysis of studies investigating the association between a traditional Mediterranean diet and depressive outcomes. Estimates are ORs, RRs or HRs of depression for people with highest adherence compared to lowest adherence (categories or quantiles specified). MDS Mediterranean diet score, rMED relative MDS, aMED alternative MDS, T tertile, Q quintile

The combined estimate from four longitudinal studies [31, 45, 46, 48] shows that people in the highest category of adherence to a Mediterranean diet have lower odds/risk of incident depressive outcomes, with an overall estimate of 0.67; 95% confidence interval (CI): 0.55, 0.82 compared to people with lowest adherence (Fig. 1). The estimate from two studies [25, 27] were produced using linear models or generalized estimating equation and therefore not comparable to the other studies: one showed a significant inverse association over time [27], and the other study, on adolescents, found no significant association [25]. The three cross-sectional studies yielded inconsistent results.

### Healthy Eating Index (HEI)

There were three longitudinal cohort studies (United Kingdom [55], Spain [48], and France [51]) with an average 6.5 years follow-up) and four cross-sectional studies (US [50, 52] and Iran [53, 54]) that used either the HEI-2005 [50, 52], the original AHEI [55] or the AHEI-2010 [48, 51, 53, 54] (Table 1**)**. The HEI-2005 is based on the Dietary Guidelines for Americans 2005, ranges 0–100 and has 12 components, each scoring five or ten points: total fruit, whole fruit, total vegetables, dark green and orange vegetables and legumes, total grains, whole grains, dairy, meat and beans, oils, saturated fat, sodium, empty calories. The AHEI includes nine components, each with a score of up to ten points except multivitamin use (vegetables, fruit, nuts and soy protein, ratio of white to red meat, cereal fiber, trans fat, polyunsaturated-to-saturated fat ratio, duration of multivitamin use, and alcohol), for a total score ranging 2.5 to 87.5. Finally, the AHEI-2010 comprises 11 items (vegetables, fruit, nuts and legumes, red/processed meat, whole grains, trans fat, long-chain (n-3) fatty acids, polyunsaturated fat, alcohol, sugar-sweetened beverages and fruit juice, and sodium) and ranges 0–110.

**Fig. 2:**
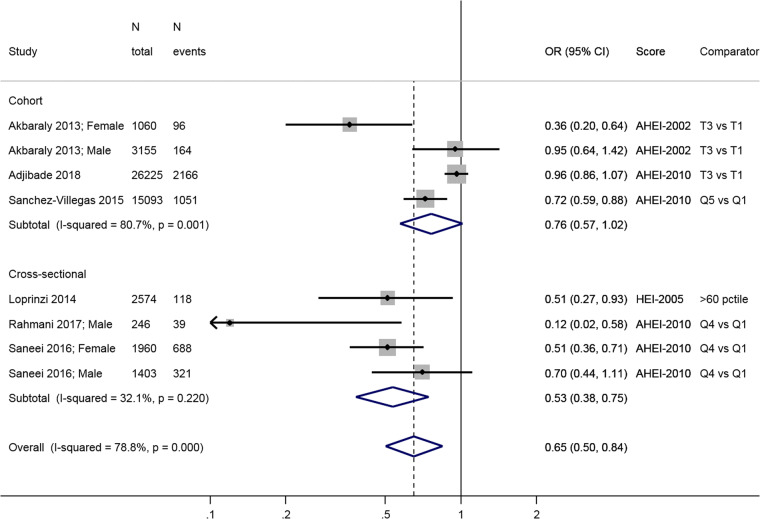
Meta-analysis of studies investigating the association between HEI/AHEI and depressive outcomes. Estimates are ORs, RRs, or HRs of depression for people with highest adherence compared to lowest adherence (categories or quantiles specified). HEI healthy eating index, AHEI Alternatative Heatlhy Eating Index, T tertile, Q5 quintile, Q4 quartile, 60pctile 60^th^ percentile

The three longitudinal studies [48, 51, 55] show a lower risk of incident depression in the high diet score category compared to low (0.76; 95% CI: 0.57, 1.02), but this association is only borderline significant at the conventional level (Fig. 2). There was large heterogeneity in the estimates of these three studies (*I*^2^ = 80.7%, *p* = 0.001). Overall, the cross-sectional studies show an inverse association between HEI-2005 or AHEI-2010 and prevalence of depression: OR = 0.53; 95% CI: 0.38, 0.75, with no apparent heterogeneity (*I*^2^ = 32.1%, *p* = 0.22) (Fig. 2).

### Dietary Approaches to Stop Hypertension (DASH)

Four studies [56–59] used the DASH diet score developed by Fung and colleagues [81] or a modified version (Table 1). It comprises eight components relative to food group intakes (negative: sweet beverages, meat, sodium; positive: fruit, vegetables, legumes and nuts, wholegrain, low-fat dairy), scores of one to five correspond to sex-specific quintiles, and the total sum score ranges 8–40.

**Fig. 3:**
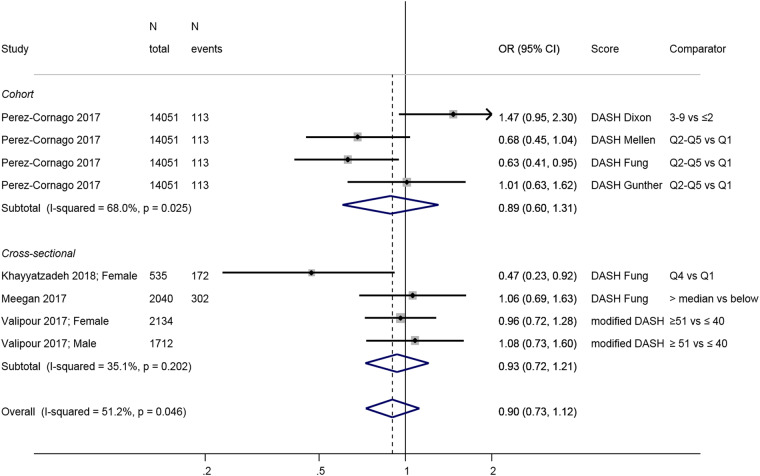
Meta-analysis of studies investigating the association between a DASH diet and depressive outcomes. Estimates are ORs, RRs, or HRs of depression for people with highest adherence compared to lowest adherence (categories or quantiles specified). DASH dietary approaches to stop hypertension, T tertile, Q5 quintile, Q4 quartile

Investigators in the only longitudinal study, the Spanish SUN cohort [58], compared the Fung DASH diet score to three other DASH scores [82–84] which use different scoring system or include nutrient intakes, and found a significant negative association with depression incidence only when using the Fung score; the other DASH scores were not associated with clinical depression (Fig. 3). Results from cross-sectional studies reveal no association with the exception of an Iranian study of adolescent girls [56] that found an inverse association between DASH and depressive symptoms. Overall, the link between adherence to a DASH diet and depression has been little studied and results are inconclusive, particularly in adults.

### Dietary Inflammatory Index (DII)

The DII is a literature-derived, population-based index that aims to quantify the overall effect of diet on inflammatory potential based on the individual inflammatory effects of up to 45 food parameters [85]. We found five cohort studies from the UK [61], the US [42], France [60], Australia [65] and Spain [67] and four cross-sectional studies from the US [62, 66], Ireland [63] and Iran [64] (Table 1).

**Fig. 4:**
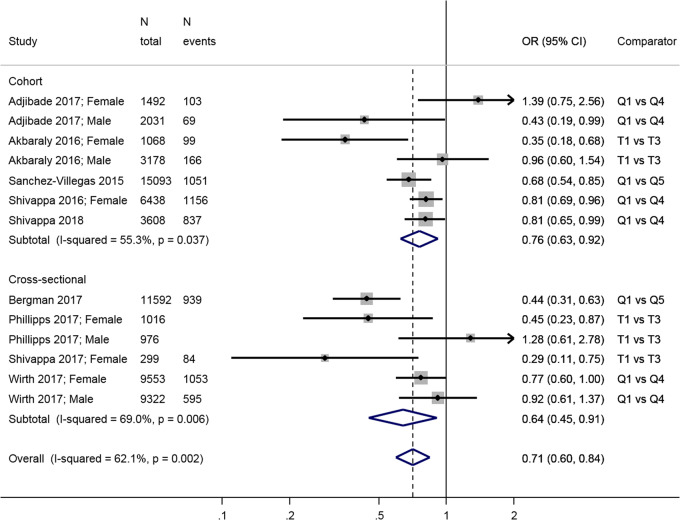
Meta-analysis of studies investigating the association between the Dietary Inflammatory Index DII and depressive outcomes. Estimates are ORs, RRs, or HRs of depression for people with lowest adherence compared to highest adherence (categories or quantiles specified). T tertile, Q5 quintile, Q4 quartile

Comparing the least inflammatory to the most inflammatory diet, there was a combined inverse association in both longitudinal (overall HR = 0.76; 95% CI: 0.63, 0.92) and cross-sectional (overall OR = 0.64; 95% CI: 0.45, 0.91) (Fig. 4) analyses. There was significant heterogeneity in the results from both longitudinal (*I*^2^ = 55.3%, *p* = 0.04) and cross-sectional studies (*I*^2^ = 69.0%, *p* = 0.006), in particular due to differences between estimates in men and women, with three studies showing a negative association in women but no relationship in men [61, 63, 66]; another study found the reverse [60].

### Other dietary indices

**Fig. 5:**
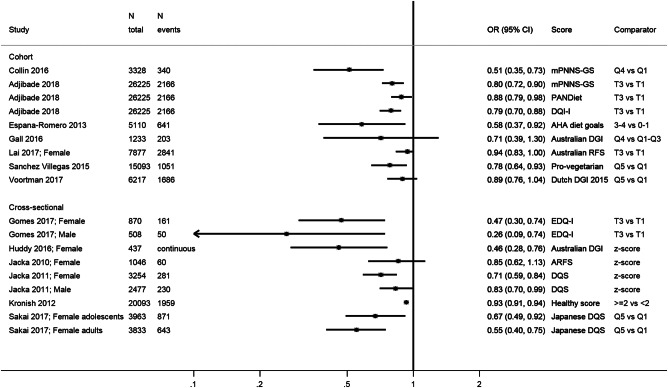
Summary of studies investigating the association between various other diet quality scores and depressive outcomes. mPNNS-GS modified score of adherence to the French dietary guidelines (PNNS), AHA American Heart Association, (A)RFS (Australian) Recommended Food Score, DGI Dietary Guidelines Index, DQI-I Diet Quality Index International, DQS Diet quality score, EDQ-I Elderly Dietary Quality Index, PANDiet Diet Quality Index Based on the Probability of Adequate Nutrient Intake, T tertile, Q5 quintile, Q4 quartile

A variety of other scores were used to describe adherence to national dietary guidelines [44, 46, 51, 69, 71, 73, 75, 77, 79], to the American Heart Association recommendations [70], and pro-vegetarian [67] or general “diet quality” scores [2, 72, 74, 76, 78] (Table 1). Owing to an absence of comparability, we show all estimates on a summary plot (Fig. 5) but do not provide an overall estimate. We observed a trend towards an inverse association between higher diet quality and depression.

### Risk of bias

We present the contour-enhanced funnel plots for the four main dietary scores on Supplemental Fig. 2. There was little evidence of publication bias as evidenced by visual inspection of the plots: estimates from the included studies are distributed equally around the overall estimate for each index used, and studies with both significant and non-significant estimates were included. Egger’s test for small study effects was significant only for the studies using the HEI or AHEI (*p* = 0.01), but the Begg test was non-significant (*p* = 0.39). All tests for small study effects were non-significant for Mediterranean, DASH and DII.

### Sensitivity analyses

Regarding outcome definition, when analyzing only studies on depressive symptoms outcomes (that is, excluding studies using clinical depression as outcome), the results remained similar for the MDS and DII (Supplemental Figs. 3 and 5 respectively), but the overall estimate for the HEI /AHEI based on three prospective studies was substantially attenuated (Supplemental Fig. 4): 0.74; 95% CI: 0.47, 1.18. Results on clinical depression all come from the Spanish SUN cohort, which showed strong prospective associations with the MDS, HEI, and DII scores [48, 67] but not consistent with different DASH scores [58]. In addition, Supplemental Figure 6 shows that for all other dietary scores, significant negative associations were reported with depressive symptoms, whereas the three studies that used clinical depression report non-significant associations [71, 75, 79]. Regarding study quality, all cross-sectional studies using HEI/AHEI were judged of low quality; therefore, when limiting the evidence to high quality studies, this dietary score only shows a weak overall estimate from three cohort studies. When assessing the differences by geographical region, we found that studies in middle-income country were all conducted in Iran. Two of them, carried out in adults, assessed the cross-sectional association between the HEI and depressive symptoms and reported similar estimates to those observed in an Irish cross-sectional study using the HEI score too. In addition, three Iranian studies were carried out on adolescents and found significant inverse associations between Mediterranean diet, DASH and DII scores and depressive symptoms. The global estimates between each of these dietary scores and depressive symptoms were not altered after excluding studies conducted in adolescents as illustrated in Supplemental Figure 7, 8 and 9, showing results for the Mediterranean diet, DASH, and DII scores respectively. Finally, having excluded the study using psychological distress [31], the overall results of the association between a Mediterranean diet and depressive outcomes remained essentially unchanged (Supplemental Figure 10).

## Discussion

### Main findings

By focusing on dietary indices, this systematic review is the first to provide an exhaustive overview of the evidence linking a wide range of comparable a priori diet quality indices and depressive outcomes. From analyses of longitudinal studies, there is a robust association between both higher adherence to a Mediterranean diet and lower adherence to a pro-inflammatory diet and a lower risk of depression. While there are fewer studies, the same trend seems apparent for indices such as the Healthy Eating Index and several other country-specific dietary guidelines scores.

### Comparison with the literature

According to recent reviews, the available evidence suggests an inverse association between healthy or prudent dietary patterns and depression, despite some inconsistent results and heterogeneity in the methods used to define healthy dietary patterns [16–20]. The beneficial effect of the Mediterranean diet has been reviewed in 2013 [21] but these studies were, with one exception, cross-sectional. The addition of recent longitudinal studies reinforces the previous review in concluding that highest compared to lowest adherence to a Mediterranean diet is associated with lower risk of incident depressive outcome. A unique feature of the present review is the inclusion and comparison of a wide range of a priori dietary scores. We found that the measures of adherence to dietary guidelines that have been commonly studied in relation to other chronic diseases and mortality, HEI and AHEI [86–88], also show encouraging results in relation to depression; however, more longitudinal studies are needed to confirm the direction of the associations.

### Biological mechanisms

As summarized in Supplemental Table 1, the dietary scores share common elements: higher fruits, vegetables, and nut intake, lower intakes of pro-inflammatory food items such as processed meats and trans fats, and alcohol in moderation. To date, a number of factors have been proposed to cause diet-induced damage to the brain, including oxidative stress, insulin resistance, inflammation, and changes in vascularization, as all these factors can be modified by dietary intake and have been associated with occurrence of depression [89]. Moreover, recent human studies [90] support extensive pre-clinical research [91], suggesting an impact of diet on the hippocampus. Fruit, vegetables, nuts, and wine in moderation have been associated with better metabolic health outcomes [92, 93], which share a common etiology with depression [94]. Those foods have antioxidant and anti-inflammatory properties. Protection against oxidation can reduce neuronal damage due to oxidative stress [17]. Systemic inflammation can affect the brain by active transport of cytokines through the brain endothelium or activation of vagal fibers, and also plays a role in the regulation of emotions through mechanisms involving neurotransmitters including serotonin, dopamine, noradrenaline, and glutamate [60]. Our results show a consistent association between an inflammatory diet (measured by the DII) and incident depressive outcomes, which supports the hypothesis that avoiding pro-inflammatory foods in favor to anti-inflammatory diet might contribute to prevent incidence of depression and depressive symptoms. Finally, an extensive body of evidence now points to the microbiome-gut-brain axis as playing a key role in neuropsychiatry, and to the primacy of diet as a factor modulating this axis [95].

### Limitations

With depression being the psychiatric disorder incurring the largest societal costs in Europe, our study is part of an effort to gather evidence on the role of nutrition in depression, to help develop recommendations and guide future psychiatric health care. Recent reviews [16–19] paved the way and our results come to complement the previous studies by focusing on dietary indices that can have a direct application in clinical settings.

However, there are various methodological considerations that need to be taken into account when interpreting the results of this meta-analysis. First, despite defining strictly our outcome of interest to unipolar depression or depressive symptoms, there was heterogeneity across studies: most used questionnaires, in particular the CES-D, although differing versions, and some questionnaires were only used in a single study (MFQ [25], BDI [56]). Only a minority of studies examined clinical depression [48, 58, 67, 71, 75, 79], assessed by clinical interview or self-reported physician diagnosis, complemented by the use of anti-depressants. Therefore, the accuracy of the prevalence or incidence may differ from one study to another. Moreover, depressive symptoms may be transient and are potentially reversible, whereas diagnosed depression is of greater severity, although they can also be reversible and transient. Our results show that most studies focused on depressive symptoms, but there is a lack of evidence for clinical depression. The only studies included in the present review that used formal diagnosis of depression as an outcome are from the SUN cohort, which showed strong and robust associations with four different dietary indices [48, 67] except for DASH scores [58], and three other studies that found no significant association with the Australian [71, 75] or with the Dutch [79] dietary guidelines scores. The meta-analysis conducted by Molendijk et al. found that there was no overall association between adherence to healthy dietary patterns and incidence of depression using a formal diagnosis as outcome [16], but this review included only three studies, one of which used internalizing disorder (not specifically depression) in children as outcome, so the comparability of these three estimates is problematic.

Second, even under the same diet index name, the operationalization differed between studies depending on the dietary data available and how they were collected. It is common for large observational studies to collect self-reported dietary data with imperfect instruments such as food frequency questionnaires. These are associated with substantial measurement error, which can reduce the ability to detect associations [96]. Moreover, the majority of studies assessed diet at a single time point and did not take into account possible changes in diet quality over time that may be concomitant to the development of depressive symptoms. Furthermore, most studies compare extreme categories, e.g., top vs. bottom, but some used tertiles, others quartiles, or quintiles, making the comparisons less straightforward. In contrast to other meta-analyses [16, 17], we presented global estimates of studies assessing the same dietary score, analyzed as categorical variable, and did not include studies assessing dietary scores as continuous variables, in order to provide more homogeneous results and an accurate quantification of the diet-depression relationship. Additionally, by presenting separate estimates according to the cross-sectional and longitudinal design of studies with long-term follow-up that should preclude reverse causality, and finding comparable results in both designs for most indices, our study provides support for an effect of overall diet on depression outcomes.

Third, we only included studies that used statistical adjustment (as opposed to simple mean comparisons between groups for instance). However, given the heterogeneity in the assessment of covariates, the comparability between studies is limited. All studies took age, sex (when relevant), energy intake and sociodemographic factors into account and the majority also included lifestyle factors and cardiometabolic markers; however, many of these used heterogeneous measures: some only adjusted for BMI, whereas others had a full cardiometabolic profile (blood pressure, cholesterol, diabetes, etc.). Also, a few studies did not include smoking nor physical activity [27, 52, 71, 76], which are common correlates to diet quality and therefore the “effect” of diet quality observed in these studies may be a proxy of a healthy overall lifestyle. However, most studies do adjust for other health behaviors and the weight of the data suggests that the relationship between diet quality and depression is independent of other health behaviors, as well as income, education, and body weight. The majority of longitudinal analyses investigated incident depression, i.e., excluded prevalent depression at baseline, except three that only adjusted for baseline symptoms/use of antidepressants [25, 46, 71]. Some cross-sectional studies included anti-depressant use or history of depression as covariates [44, 54, 57, 59, 63, 78], but no clear trend was observed in terms of effect size or significance of the estimates produced when comparing these with the studies that did not.

Fourth, the vast majority of the studies included in this systematic review were conducted in high income countries (Australia, France, Greece, Ireland, Japan, Netherlands, Norway, Spain, UK, US), with only seven studied in low-and-middle income countries (Brazil [72] and Iran [49, 53, 54, 56, 59, 64]. Hence, the generalizability of the findings to low-and-middle income countries is limited. Moreover, we did not want to include an age limit and our systematic review includes three studies on adolescents [25, 49, 64]. Psychological disorders may express differently during adolescence as opposed to later in life, but the exclusion of those studies did not change the overall results and conclusions. Therefore, considering all the above limitations, extra care should be applied when using and interpreting the meta-analysis estimates.

## Conclusions

Our review shows that there is observational evidence to suggest that both adhering to a healthy diet, in particular a traditional Mediterranean diet, and avoiding a pro-inflammatory diet is associated with reduced risk of depressive symptoms or clinical depression. That the majority of recovered studies were cross-sectional in design, with the problem of reverse causality being acute in the context of diet and depression, there is a clear need for more prospective studies. Moreover, while recent intervention studies provide preliminary evidence [97, 98], further well-powered clinical trials are required to assess the role of dietary patterns in the prevention of onset, severity, and recurrence of depressive episodes.

## Electronic supplementary material

Supplemental material
