# Correction: Healthy dietary indices and risk of depressive outcomes: a systematic review and meta-analysis of observational studies

**DOI:** 10.1038/s41380-018-0299-7

**Published:** 2018-11-21

**Authors:** Camille Lassale, G. David Batty, Amaria Baghdadli, Felice Jacka, Almudena Sánchez-Villegas, Mika Kivimäki, Tasnime Akbaraly

**Affiliations:** 1 Department of Epidemiology and Public Health, University College London, London, WC1E 6BT United Kingdom; 2 Department of Behavioural Science and Health, University College London, London, WC1E 6BT United Kingdom; 3 Department of Psychiatry & Autism Resources Centre, University Hospital of Montpellier, CHRU de Montpellier, F-34000 France; 4 INSERM, U1018, Centre for Research in Epidemiology and Population Health, Hôpital Paul Brousse, Villejuif, France; 5 Deakin University, Food & Mood Centre, IMPACT Strategic Research Centre, School of Medicine, Barwon Health, Geelong, Australia; 6 Nutrition Research Group, Research Institute of Biomedical and Health Sciences, University of Las Palmas de Gran Canaria, Las Palmas de Gran Canaria, Spain; 7 Ciber de Fisiopatología de la Obesidad y Nutrición (CIBER OBN), Instituto de Salud Carlos III, Madrid, Spain; 8 Clinicum, Faculty of Medicine, University of Helsinki, Helsinki, Finland; 9 MMDN, University of Montpellier, EPHE, INSERM, U1198, Montpellier, F-34095 France

Correction to: *Molecular Psychiatry*; 10.1038/s41380-018-0237-8; published online 26 September 2018.

This article was originally published under standard licence, but has now been made available under a CC BY 4.0 license. The PDF and HTML versions of the paper have been modified accordingly.

