# Supplementary material for: Healthy dietary indices and risk of depressive outcomes: a systematic review and meta-analysis of observational studies
Source: Mol Psychiatry. 2018 Sep 26;24(7):965–86. doi: 10.1038/s41380-018-0237-8 (PMC6755986; doi:10.1038/s41380-018-0237-8)
Supplement: Supplementary file 1 — Supplemental material [file 41380_2018_237_MOESM1_ESM.docx]

**Supplemental Figure 1.** Publication selection process, systematic review of studies of a priori diet quality indices and depression risk


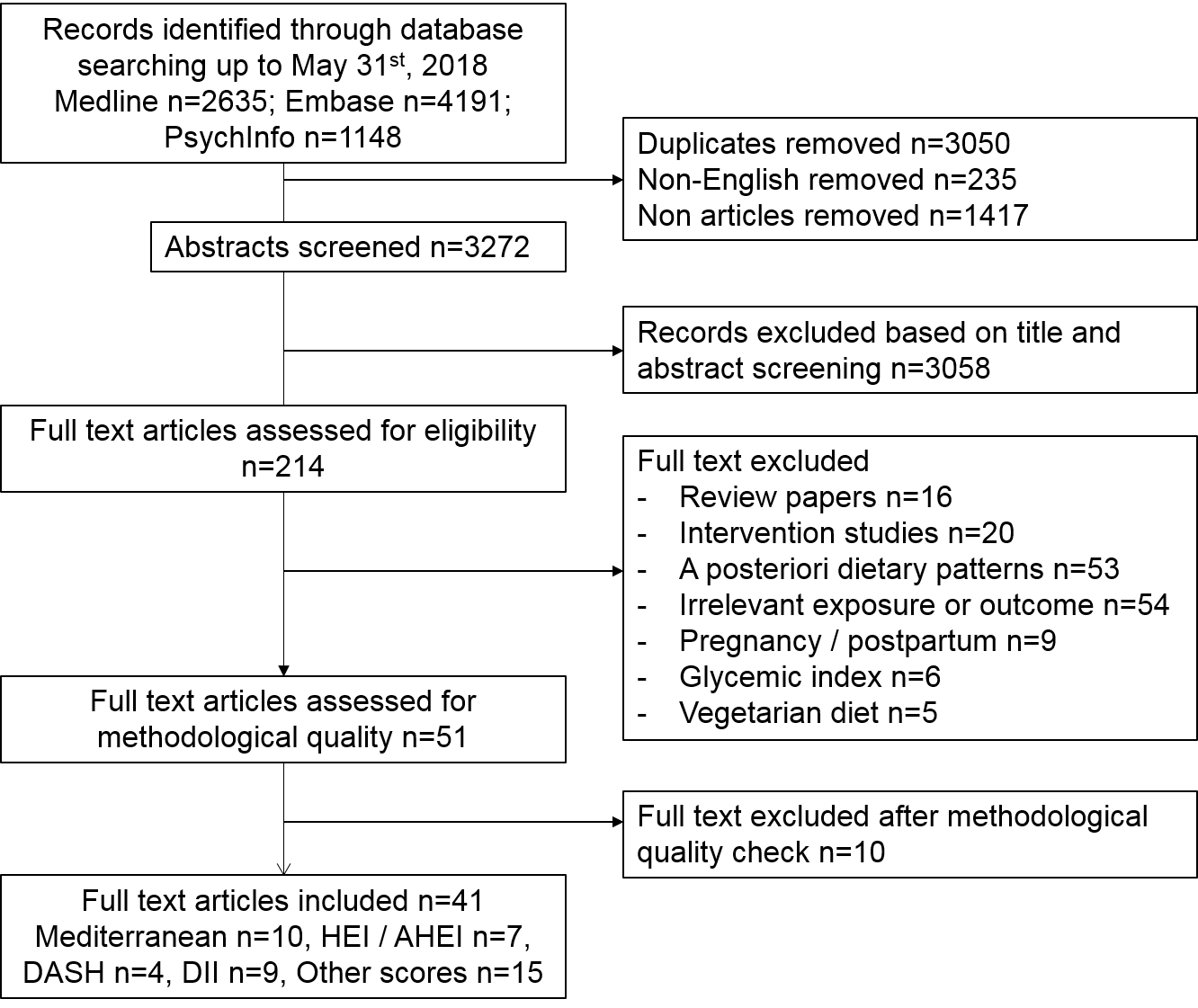


Abbreviations: HEI, healthy eating index; AHEI, alternative Healthy Eating Index; DASH, dietary approaches to stop hypertension; DII, dietary inflammatory index

**Supplemental Figure 2.** Contour-enhanced funnel plots of the studies that assessed the relationship between four main dietary scores and depression, by score.


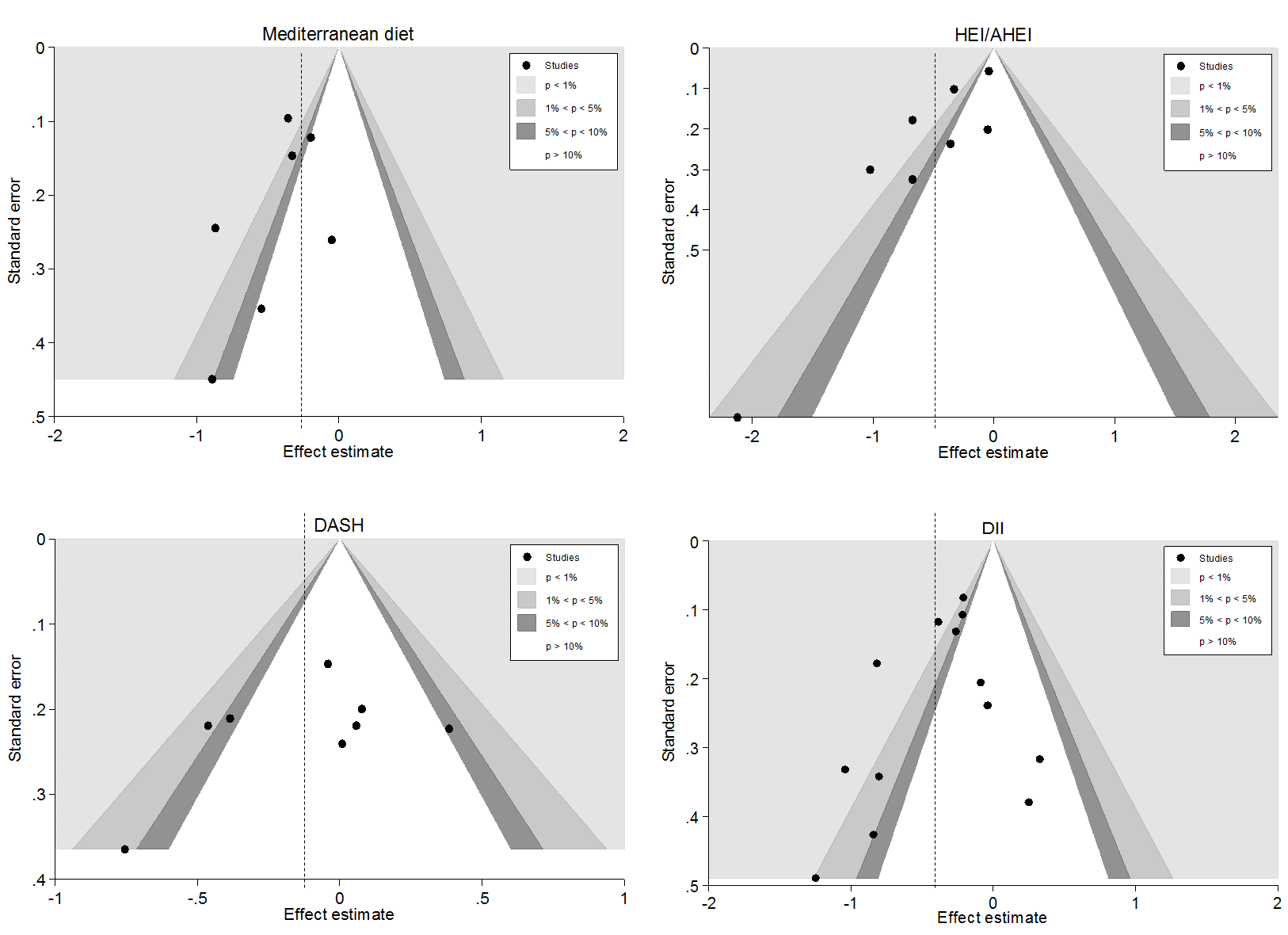


The dotted line is the overall estimate. Abbreviations: DASH, dietary approaches to stop hypertension; HEI, healthy eating index; DII, dietary inflammatory index

**Supplemental Figure 3.** Meta-analysis of studies investigating the association between a traditional Mediterranean diet and depressive symptoms outcomes assessed by questionnaire (i.e. excluding the SUN cohort).


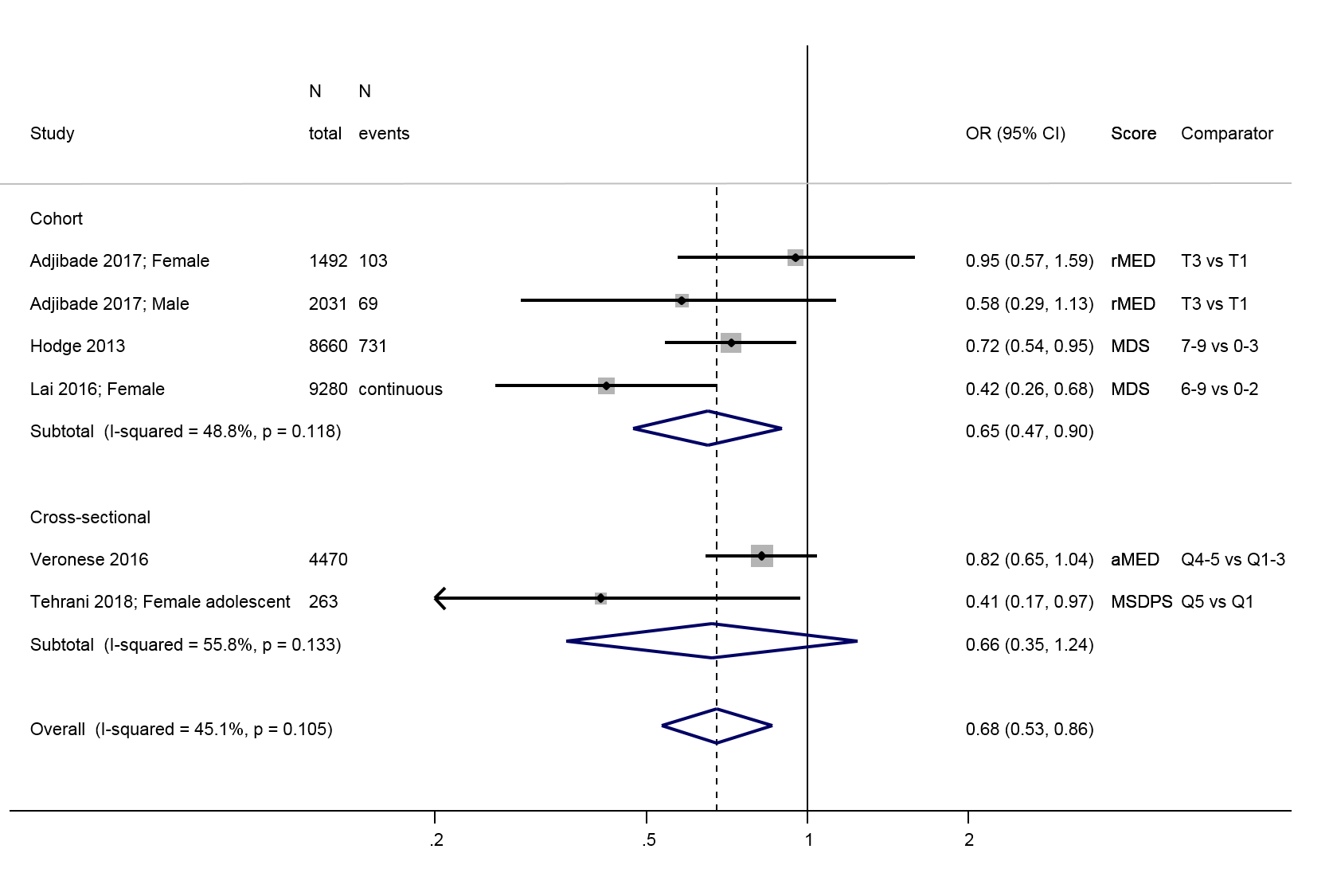


**Supplemental Figure 4.** Meta-analysis of studies investigating the association between the HEI/AHEI score and depressive symptoms outcomes assessed by questionnaire (i.e. excluding the SUN cohort)


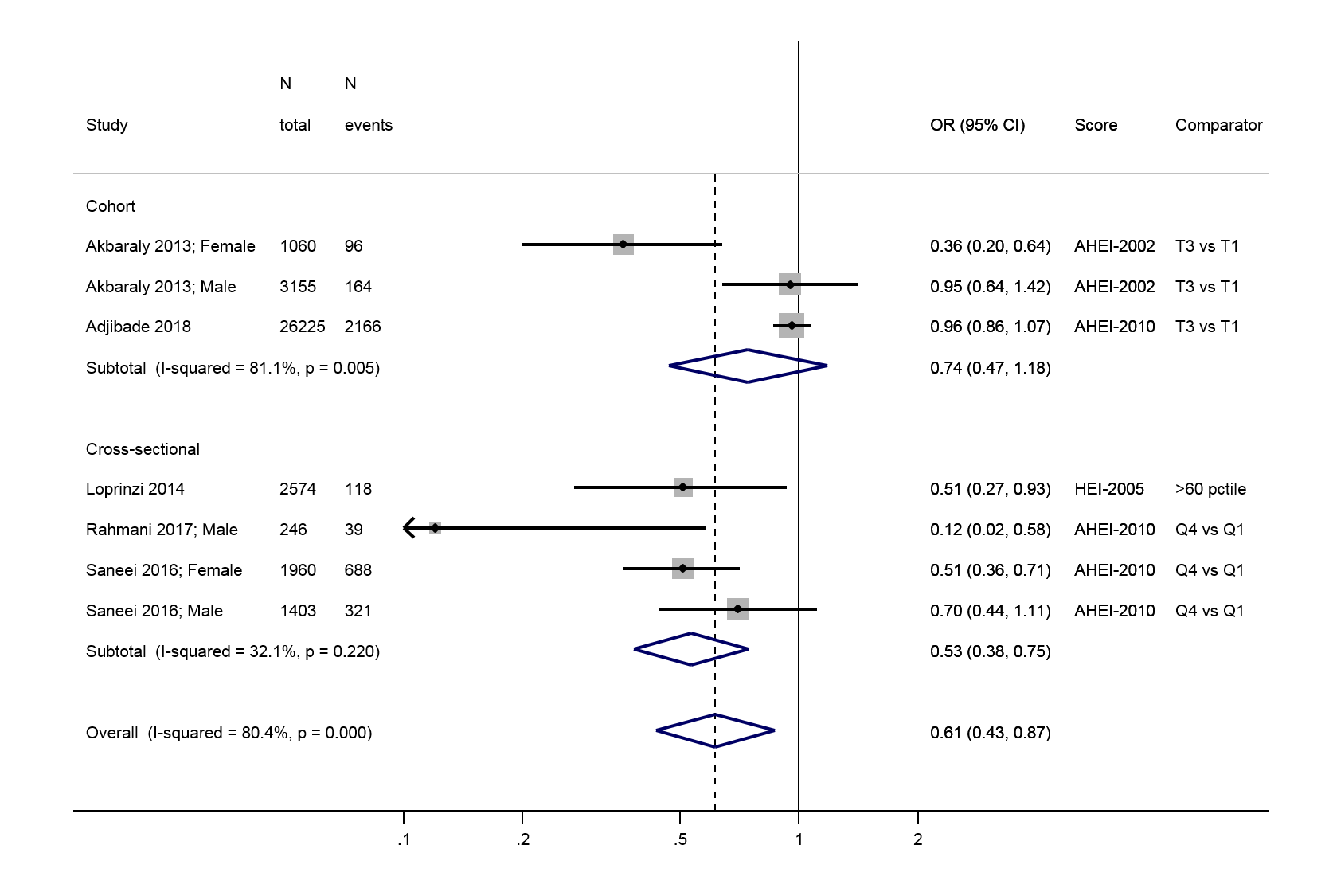


**Supplemental Figure 5.** Meta-analysis of studies investigating the association between the Dietary Inflammatory Index DII and depressive symptoms outcomes assessed by questionnaire (i.e. excluding the SUN cohort)


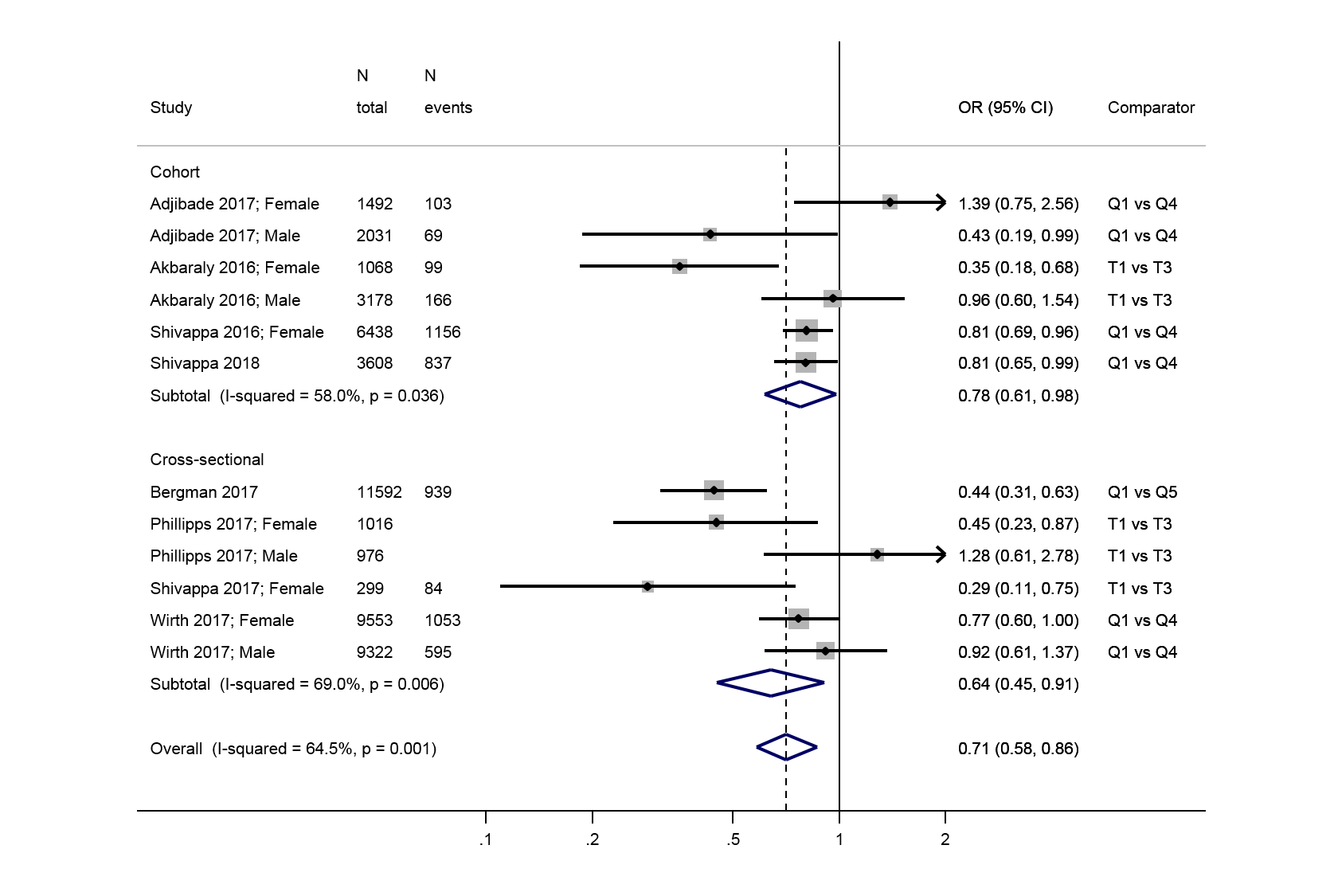


**Supplemental Figure 6**. Summary of studies investigating the association between various other diet quality scores and depressive symptoms outcomes assessed by questionnaire

**
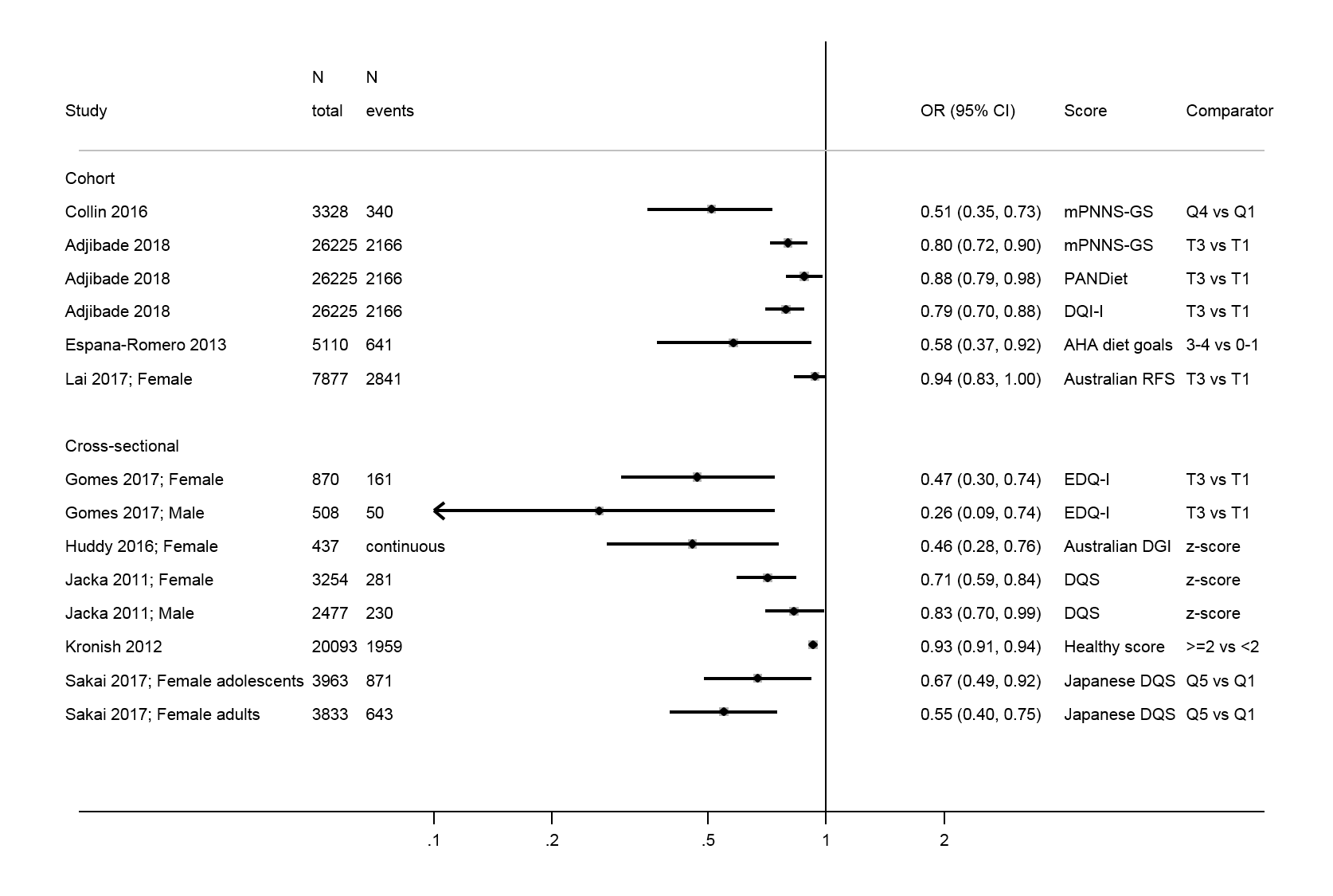
**

**Supplemental Figure 7.** Meta-analysis of studies investigating the association between a traditional Mediterranean diet and depressive outcomes in adults only (exclusion of Iranian adolescent study)**
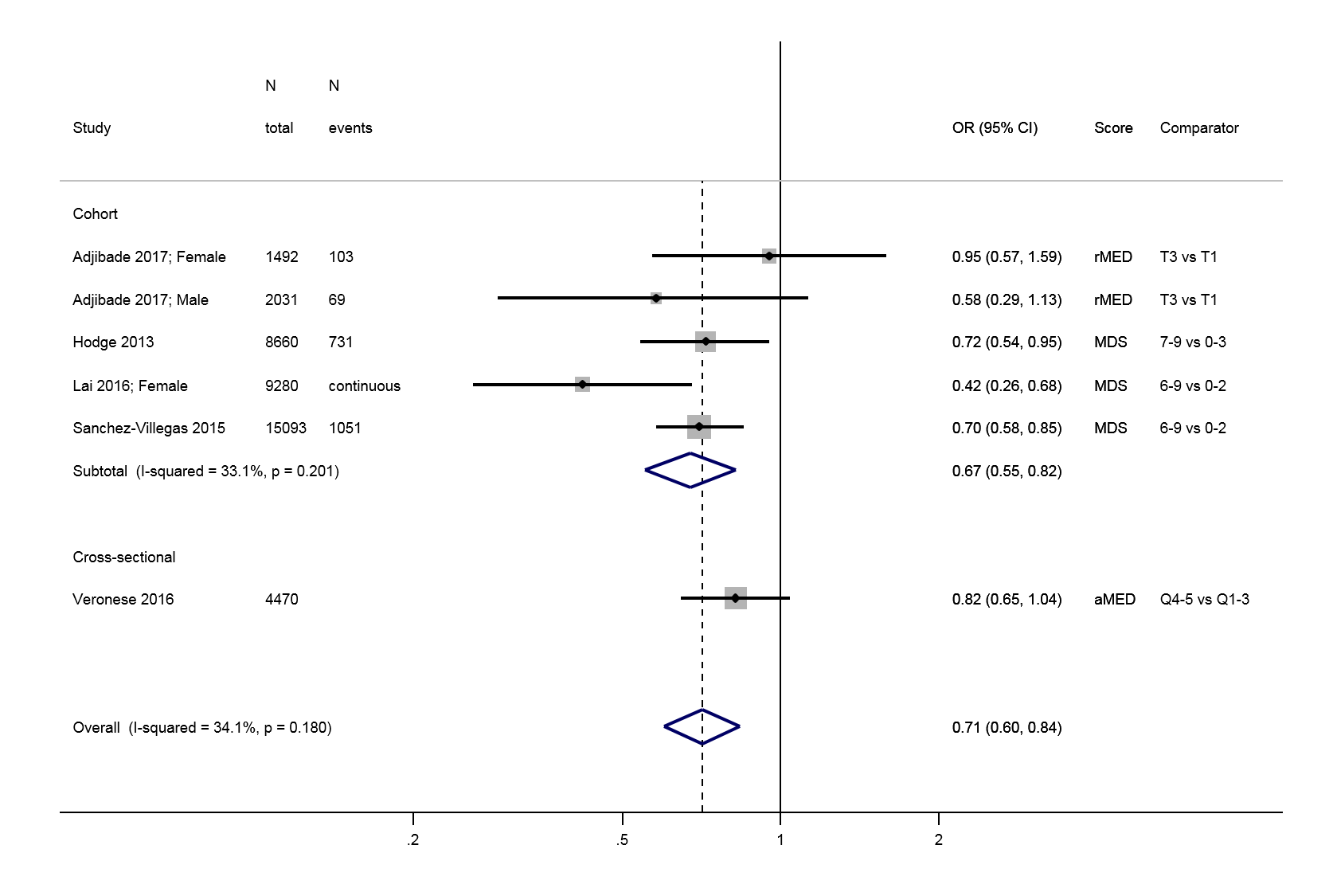
**

**Supplemental Figure 8.** Meta-analysis of studies investigating the association between the DASH diet and depressive outcomes in adults only (exclusion of Iranian adolescent study)**
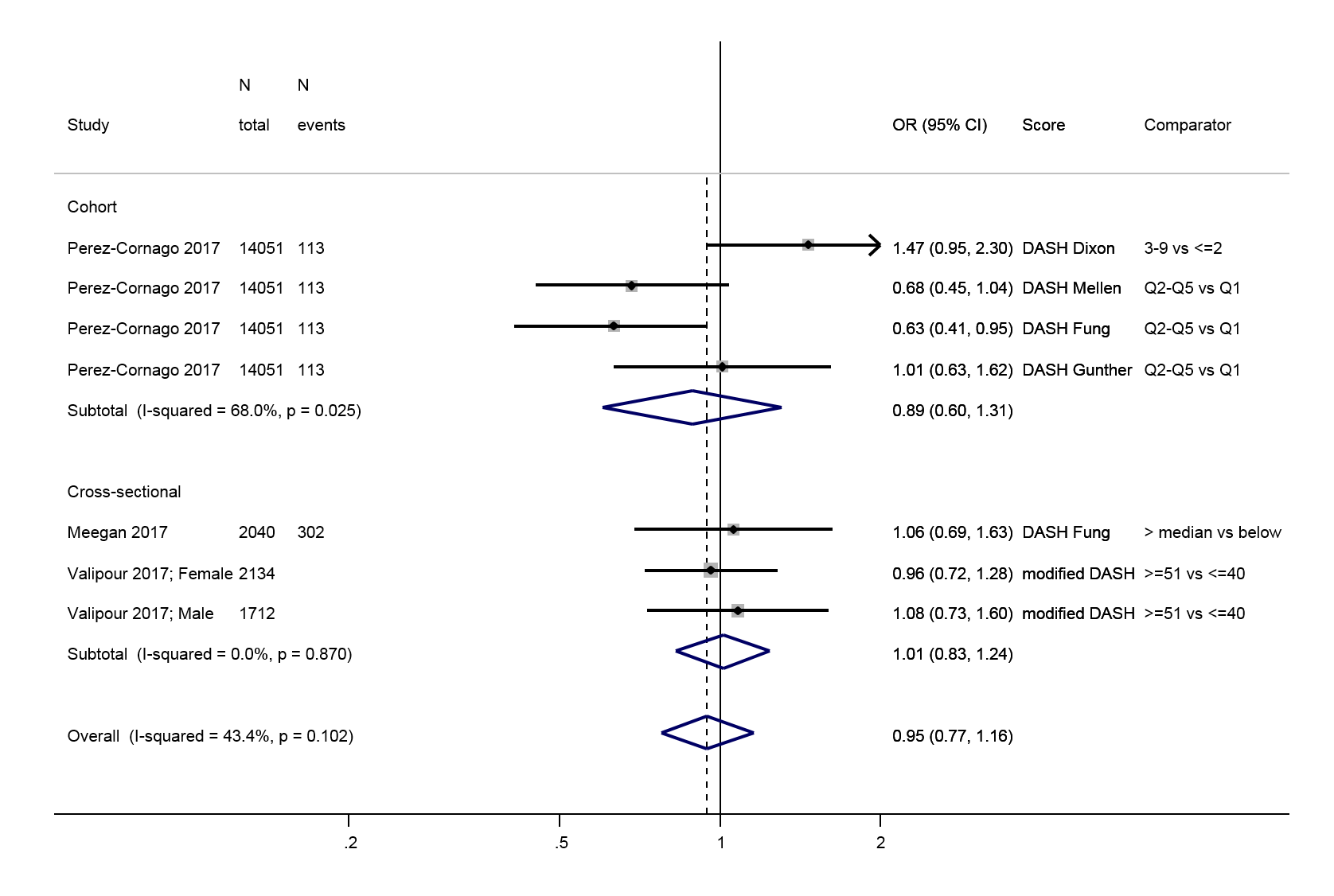
**

**Supplemental Figure 9**. Meta-analysis of studies investigating the association between the Dietary Inflammatory Index DII and depressive outcomes in adults only (exclusion of Iranian adolescent study)

**
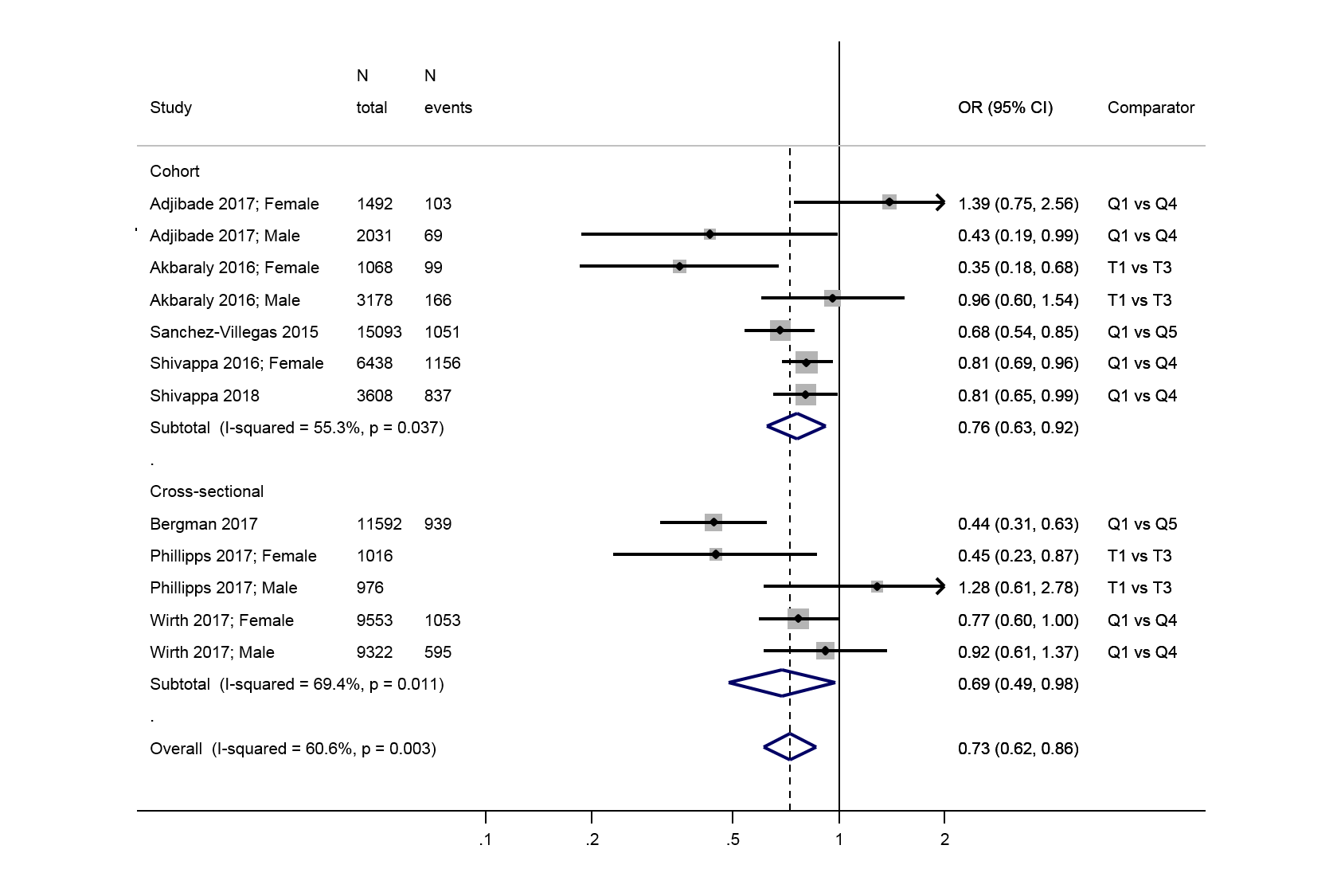
**

**Supplemental Figure 10.** Meta-analysis of studies investigating the association between a traditional Mediterranean diet and depressive outcomes after exclusion of the study on psychological distress

**
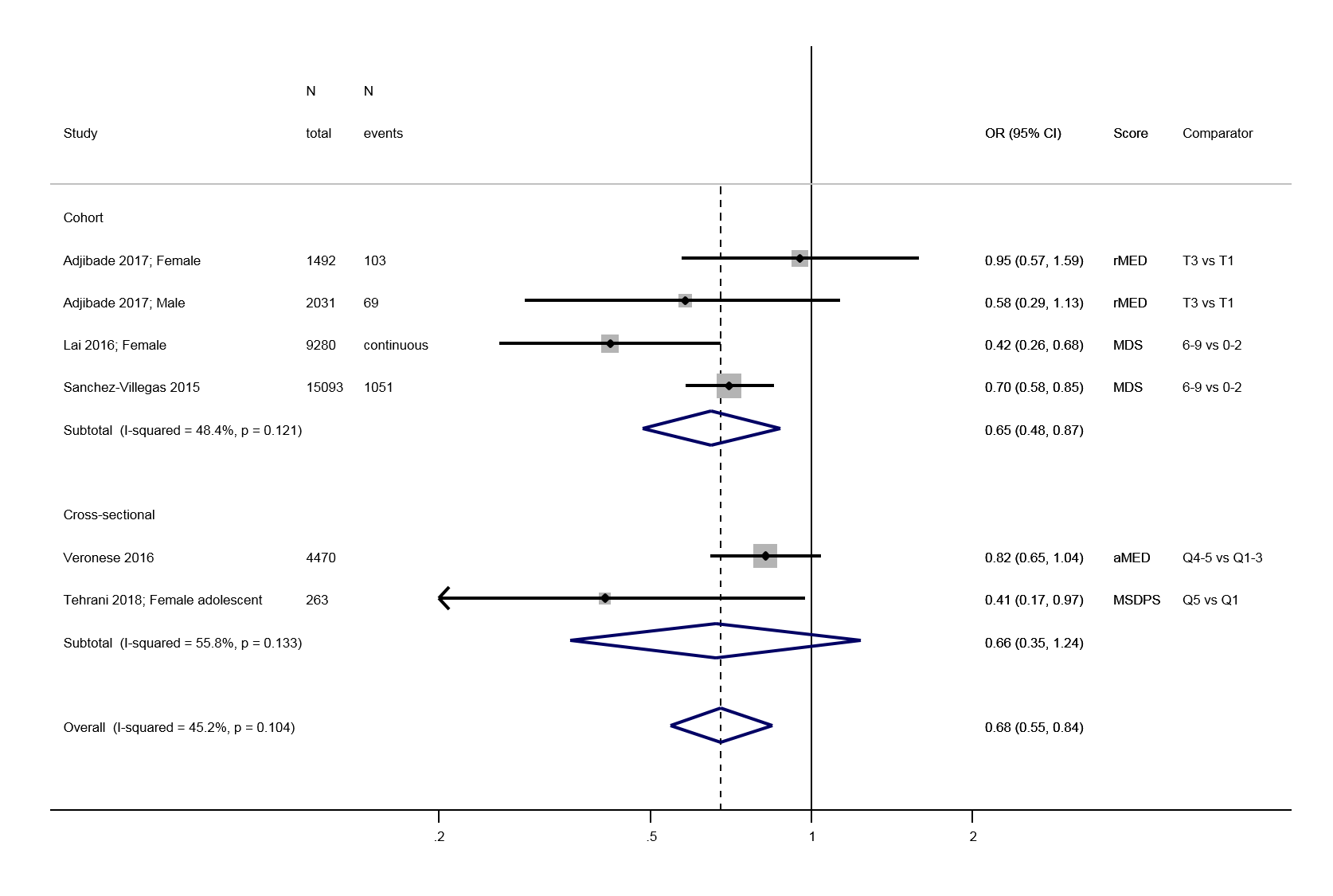
**

**Supplemental Table 1.** Food groups and nutrients included in the calculation of the main scores used in the literature in relation to depression

|  | **MDS** | **rMED** | **aMED** | **MSDPS** | **HEI-2005** | **AHEI original** | **AHEI-2010** | **DASH (Fung)** | **DASH (Mellen)** |
| --- | --- | --- | --- | --- | --- | --- | --- | --- | --- |
| **Food** |  |  |  |  |  |  |  |  |  |
| Alcohol | + (moderate) | + (moderate) | + (moderate) | + (moderate) | - | + (moderate) | + (moderate) |  |  |
| Dairy | - | - | - | - | + |  |  | + (low-fat) |  |
| Egg |  |  |  | - |  |  |  |  |  |
| Empty calorie food | |  |  |  | - |  |  |  |  |
| Fish | + | + | + | + | + |  |  |  |  |
| Fruit | + | + | + | + | + | + | + | + |  |
| Grains | + | + | + |  | + |  |  |  |  |
| Refined |  |  |  |  |  |  |  |  |  |
| Whole |  |  |  | + | + |  | + | + |  |
| Legumes | + | + | + | + | + | + (soy) | + | + |  |
| Nuts | + | + |  | + |  | + | + | + |  |
| Olive oil |  | + | + | + | + (any oil) |  |  |  |  |
| Meat |  |  |  |  | + (with beans) |  |  | - |  |
| Poultry |  |  | - | - |  | + |  |  |  |
| Red and processed | - | - | - | - |  | - | - |  |  |
| Potatoes |  |  | + | + |  |  |  |  |  |
| Sugar sweetened beverages / sweet products | | | | - | - |  | - | - |  |
| Solid fat |  |  |  |  | - |  |  |  |  |
| Total protein foods | |  |  |  | + |  |  |  |  |
| Vegetables (no potatoes) | + | + | + | + | + | + | + | + |  |
| **Nutrients** |  |  |  |  |  |  |  |  |  |
| Dietary fiber | |  |  |  |  | + |  |  | + |
| Protein |  |  |  |  |  |  |  |  | + |
| Fat |  |  |  |  |  |  |  |  | - |
| PUFA |  |  |  |  |  | + | + |  |  |
| MUFA | + |  |  |  |  |  |  |  |  |
| SFA | - |  |  |  | - | - |  |  | - |
| Cholesterol | |  |  |  |  |  |  |  | - |
| Trans-fat |  |  |  |  |  | - | - |  |  |
| Calcium |  |  |  |  |  |  |  |  | + |
| Potassium |  |  |  |  |  |  |  |  | + |
| Magnesium | |  |  |  |  |  |  |  | + |
| Sodium |  |  |  |  | - |  | - | - | - |
| Vitamin use | |  |  |  |  | + |  |  |  |

The (+) represents a “beneficial” component, i.e. higher intake is scored high. The (-) represents a “detrimental” component, i.e. higher intake is scored low. If specified “moderate”, the scoring is following a bell-shape, i.e. both very low and very high intake are scored low, and an intermediate intake is scored high. In yellow are highlighted the elements that are common to most of the scores. The grey areas are components that are common to most of the scores described.

Abbreviations: **MDS**, Mediterranean Diet Score; **rMED**, relative Mediterranean Diet Score; **aMED**, alternative Mediterranean Diet Score; **DASH**, dietary approaches to stop hypertension; **HEI**, healthy eating index; **AHEI**, alternative healthy eating index; **DII**, dietary inflammatory index

**Supplemental material: List of papers excluded by primary reason**

**Reason**: Irrelevant exposure or outcome, direction of the relationship assessed

Abshire DA, Lennie TA, Chung ML, Biddle MJ, Barbosa-Leiker C, Moser DK. 2017. Body Mass Index Category Moderates the Relationship Between Depressive Symptoms and Diet Quality in Overweight and Obese Rural-Dwelling Adults. J Rural Health 07:07.

Al Rifai M, Greenland P, Blaha MJ, Michos ED, Nasir K, Miedema MD, Yeboah J, Sandfort V, Frazier-Wood AC, Shea S and others. 2018. Factors of health in the protection against death and cardiovascular disease among adults with subclinical atherosclerosis. Am Heart J 198:180-188.

Antonogeorgos G, Panagiotakos DB, Pitsavos C, Papageorgiou C, Chrysohoou C, Papadimitriou GN, Stefanadis C. 2012. Understanding the role of depression and anxiety on cardiovascular disease risk, using structural equation modeling; the mediating effect of the Mediterranean diet and physical activity: the ATTICA study. Ann Epidemiol 22(9):630-637.

Appelhans BM, Whited MC, Schneider KL, Ma Y, Oleski JL, Merriam PA, Waring ME, Olendzki BC, Mann DM, Ockene IS and others. 2012. Depression severity, diet quality, and physical activity in women with obesity and depression. J Acad Nutr Diet 112(5):693-698.

Assmann KE, Adjibade M, Andreeva VA, Hercberg S, Galan P, Kesse-Guyot E. 2018a. Association Between Adherence to the Mediterranean Diet at Midlife and Healthy Aging in a Cohort of French Adults. J Gerontol A Biol Sci Med Sci 73(3):347-354.

Assmann KE, Adjibade M, Shivappa N, Hebert JR, Wirth MD, Touvier M, Akbaraly T, Hercberg S, Galan P, Julia C and others. 2018b. The Inflammatory Potential of the Diet at Midlife Is Associated with Later Healthy Aging in French Adults. Journal of Nutrition 148(3):437-444.

Assmann KE, Andreeva VA, Camilleri GM, Verger EO, Jeandel C, Hercberg S, Galan P, Kesse-Guyot E. 2016. Dietary scores at midlife and healthy ageing in a French prospective cohort. Br J Nutr 116(4):666-676.

Betancourt J, Rios JL, Pagan I, Fabian C, Gonzalez AM, Cruz SY, Gonzalez MJ, Rivera WT, Palacios C. 2013. Non-medical use of prescription drugs and its association with socio-demographic characteristics, dietary pattern, and perceived academic load and stress in college students in Puerto Rico. P R Health Sci J 32(2):89-94.

Beydoun MA, Fanelli-Kuczmarski MT, Shaked D, Dore GA, Beydoun HA, Rostant OS, Evans MK, Zonderman AB. 2016. Alternative Pathway Analyses Indicate Bidirectional Relations between Depressive Symptoms, Diet Quality, and Central Adiposity in a Sample of Urban US Adults. Journal of Nutrition 146(6):1241-1249.

Beydoun MA, Kuczmarski MT, Mason MA, Ling SM, Evans MK, Zonderman AB. 2009. Role of depressive symptoms in explaining socioeconomic status disparities in dietary quality and central adiposity among US adults: a structural equation modeling approach. American Journal of Clinical Nutrition 90(4):1084-1095.

Beydoun MA, Shroff MR, Beydoun HA, Zonderman AB. 2010. Serum folate, vitamin B-12, and homocysteine and their association with depressive symptoms among U.S. adults. Psychosom Med 72(9):862-873.

Bonnet F, Irving K, Terra JL, Nony P, Berthezene F, Moulin P. 2005a. Anxiety and depression are associated with unhealthy lifestyle in patients at risk of cardiovascular disease. Atherosclerosis 178(2):339-344.

Bonnet F, Irving K, Terra JL, Nony P, Berthezene F, Moulin P. 2005b. Depressive symptoms are associated with unhealthy lifestyles in hypertensive patients with the metabolic syndrome. J Hypertens 23(3):611-617.

Crichton GE, Bryan J, Hodgson JM, Murphy KJ. 2013. Mediterranean diet adherence and self-reported psychological functioning in an Australian sample. Appetite 70:53-59.

Florez KR, Dubowitz T, Ghosh-Dastidar MB, Beckman R, Collins RL. 2015. Associations between depressive symptomatology, diet, and body mass index among participants in the supplemental nutrition assistance program. J Acad Nutr Diet 115(7):1102-1108.

Gopinath B, Flood VM, Kifley A, Louie JC, Mitchell P. 2016. Association Between Carbohydrate Nutrition and Successful Aging Over 10 Years. J Gerontol A Biol Sci Med Sci 71(10):1335-1340.

Grossniklaus DA, Dunbar SB, Tohill BC, Gary R, Higgins MK, Frediani J. 2010. Psychological factors are important correlates of dietary pattern in overweight adults. J Cardiovasc Nurs 25(6):450-460.

Hansson E, Daukantaite D, Johnsson P. 2016. Typical patterns of disordered eating among Swedish adolescents: associations with emotion dysregulation, depression, and self-esteem. J Eat Disord 4:28.

Hoare E, Dash SR, Varsamis P, Jennings GL, Kingwell BA. 2017. Fasting Plasma Glucose, Self-Appraised Diet Quality and Depressive Symptoms: A US-Representative Cross-Sectional Study. Nutrients 9(12):07.

Hoerster KD, Wilson S, Nelson KM, Reiber GE, Masheb RM. 2016. Diet quality is associated with mental health, social support, and neighborhood factors among Veterans. Eating Behaviors 23:168-173.

Hong SA, Peltzer K. 2017. Dietary behaviour, psychological well-being and mental distress among adolescents in Korea. Child Adolesc Psychiatry Ment Health 11:56.

Honkalampi K, Ruusunen A, Viinamaki H, Koivumaa-Honkanen H, Valkonen-Korhonen M, Lehto SM. 2017. Dietary patterns are associated with the prevalence of alexithymia. Scand J Psychol 58(4):318-323.

Hurley KM, Black MM, Merry BC, Caulfield LE. 2015. Maternal mental health and infant dietary patterns in a statewide sample of Maryland WIC participants. Matern Child Nutr 11(2):229-239.

Hurley KM, Caulfield LE, Sacco LM, Costigan KA, Dipietro JA. 2005. Psychosocial influences in dietary patterns during pregnancy. Journal of the American Dietetic Association 105(6):963-966.

Ibarra O, Gili M, Roca M, Vives M, Serrano MJ, Pareja A, Garcia-Campayo J, Gomez-Juanes R, Garcia-Toro M. 2014. The Mediterranean diet and micronutrient levels in depressive patients. Nutr Hosp 31(3):1171-1175.

Jacka FN, Pasco JA, Mykletun A, Williams LJ, Nicholson GC, Kotowicz MA, Berk M. 2011. Diet quality in bipolar disorder in a population-based sample of women. J Affect Disord 129(1-3):332-337.

Kazaz I, Angin E, Kabaran S, Iyigun G, Kirmizigil B, Malkoc M. 2018. Evaluation of the physical activity level, nutrition quality, and depression in patients with metabolic syndrome: Comparative study. Medicine (Baltimore) 97(18):e0485.

Kesse-Guyot E, Andreeva VA, Jeandel C, Ferry M, Hercberg S, Galan P. 2012a. A healthy dietary pattern at midlife is associated with subsequent cognitive performance. Journal of Nutrition 142(5):909-915.

Kesse-Guyot E, Peneau S, Ferry M, Jeandel C, Hercberg S, Galan P, Group SVMR. 2011. Thirteen-year prospective study between fish consumption, long-chain n-3 fatty acids intakes and cognitive function. J Nutr Health Aging 15(2):115-120.

Kesse-Guyot E, Touvier M, Andreeva VA, Jeandel C, Ferry M, Hercberg S, Galan P. 2012b. Cross-sectional but not longitudinal association between n-3 fatty acid intake and depressive symptoms: results from the SU.VI.MAX 2 study. Am J Epidemiol 175(10):979-987.

Kim TH, Choi JY, Lee HH, Park Y. 2015. Associations between Dietary Pattern and Depression in Korean Adolescent Girls. J Pediatr Adolesc Gynecol 28(6):533-537.

Klassen AC, Smith KC, Black MM, Caulfield LE. 2009. Mixed method approaches to understanding cancer-related dietary risk reduction among public housing residents. J Urban Health 86(4):624-640.

Kontogianni MD, Panagiotakos DB. 2014. Dietary patterns and stroke: a systematic review and re-meta-analysis. Maturitas 79(1):41-47.

Martinez-Lapiscina EH, Clavero P, Toledo E, Estruch R, Salas-Salvado J, San Julian B, Sanchez-Tainta A, Ros E, Valls-Pedret C, Martinez-Gonzalez MA. 2013. Mediterranean diet improves cognition: the PREDIMED-NAVARRA randomised trial. J Neurol Neurosurg Psychiatry 84(12):1318-1325.

McMartin SE, Kuhle S, Colman I, Kirk SF, Veugelers PJ. 2012. Diet quality and mental health in subsequent years among Canadian youth. Public Health Nutr 15(12):2253-2258.

Ntanasi E, Yannakoulia M, Kosmidis MH, Anastasiou CA, Dardiotis E, Hadjigeorgiou G, Sakka P, Scarmeas N. 2018. Adherence to Mediterranean Diet and Frailty. Journal of the American Medical Directors Association 19(4):315-322.e312.

Paans NPG, Bot M, Brouwer IA, Visser M, Roca M, Kohls E, Watkins E, Penninx B. 2018. The association between depression and eating styles in four European countries: The MooDFOOD prevention study. J Psychosom Res 108:85-92.

Pagoto SL, Ma Y, Bodenlos JS, Olendzki B, Rosal MC, Tellez T, Merriam P, Ockene IS. 2009. Association of depressive symptoms and lifestyle behaviors among Latinos at risk of type 2 diabetes. Journal of the American Dietetic Association 109(7):1246-1250.

Pogosova N, Kotseva K, De Bacquer D, von Kanel R, De Smedt D, Bruthans J, Dolzhenko M, Investigators E. 2017. Psychosocial risk factors in relation to other cardiovascular risk factors in coronary heart disease: Results from the EUROASPIRE IV survey. A registry from the European Society of Cardiology. European Journal of Preventive Cardiology 24(13):1371-1380.

Poorrezaeian M, Siassi F, Milajerdi A, Qorbani M, Karimi J, Sohrabi-Kabi R, Pak N, Sotoudeh G. 2017. Depression is related to dietary diversity score in women: a cross-sectional study from a developing country. Ann Gen Psychiatry 16:39.

Poorrezaeian M, Siassi F, Qorbani M, Karimi J, Koohdani F, Asayesh H, Sotoudeh G. 2015. Association of dietary diversity score with anxiety in women. Psychiatry Res 230(2):622-627.

Rahe C, Baune BT, Unrath M, Arolt V, Wellmann J, Wersching H, Berger K. 2015. Associations between depression subtypes, depression severity and diet quality: cross-sectional findings from the BiDirect Study. BMC Psychiatry 15:38.

Rahi B, Ajana S, Tabue-Teguo M, Dartigues JF, Peres K, Feart C. 2017. High adherence to a Mediterranean diet and lower risk of frailty among French older adults community-dwellers: Results from the Three-City-Bordeaux Study. Clinical Nutrition 31:31.

Salehi-Abargouei A, Esmaillzadeh A, Azadbakht L, Keshteli AH, Afshar H, Feizi A, Feinle-Bisset C, Adibi P. 2018. Do patterns of nutrient intake predict self-reported anxiety, depression and psychological distress in adults? SEPAHAN study. Clinical Nutrition 21:21.

Sanchez-Villegas A, Henriquez P, Bes-Rastrollo M, Doreste J. 2006. Mediterranean diet and depression. Public Health Nutr 9(8A):1104-1109.

Sanchez-Villegas A, Zazpe I, Santiago S, Perez-Cornago A, Martinez-Gonzalez MA, Lahortiga-Ramos F. 2018. Added sugars and sugar-sweetened beverage consumption, dietary carbohydrate index and depression risk in the Seguimiento Universidad de Navarra (SUN) Project. Br J Nutr 119(2):211-221.

Sepulveda A, Carrobles JA, Gandarillas AM. 2010. Associated factors of unhealthy eating patterns among Spanish university students by gender. Span J Psychol 13(1):364-375.

Sharpe PA, Whitaker K, Alia KA, Wilcox S, Hutto B. 2016. Dietary Intake, Behaviors and Psychosocial Factors Among Women from Food-Secure and Food-Insecure Households in the United States. Ethn Dis 26(2):139-146.

Suga H, Asakura K, Kobayashi S, Nojima M, Sasaki S, Three-generation Study of Women on D, Health Study G. 2018. Association between habitual tryptophan intake and depressive symptoms in young and middle-aged women. J Affect Disord 231:44-50.

Tajik E, Latiffah AL, Awang H, Siti Nur'Asyura A, Chin YS, Azrin Shah AB, Patricia Koh CH, Mohd Izudin Hariz CG. 2016. Unhealthy diet practice and symptoms of stress and depression among adolescents in Pasir Gudang, Malaysia. Obes Res Clin Pract 10(2):114-123.

Thomson JL, Tussing-Humphreys LM, Goodman MH, Olender S. 2016. Baseline Demographic, Anthropometric, Psychosocial, and Behavioral Characteristics of Rural, Southern Women in Early Pregnancy. Matern Child Health J 20(9):1980-1988.

Trudel-Fitzgerald C, Tworoger SS, Poole EM, Williams DR, Kubzansky LD. 2016. Prospective Changes in Healthy Lifestyle Among Midlife Women: When Psychological Symptoms Get in the Way. Am J Prev Med 51(3):327-335.

Voltas N, Arija V, Aparicio E, Canals J. 2016. Longitudinal study of psychopathological, anthropometric and sociodemographic factors related to the level of Mediterranean diet adherence in a community sample of Spanish adolescents. Public Health Nutr 19(10):1812-1822.

Whitaker KM, Sharpe PA, Wilcox S, Hutto BE. 2014. Depressive symptoms are associated with dietary intake but not physical activity among overweight and obese women from disadvantaged neighborhoods. Nutrition Research 34(4):294-301.

**Reason**: A posteriori dietary patterns

Akbaraly TN, Brunner EJ, Ferrie JE, Marmot MG, Kivimaki M, Singh-Manoux A. 2009. Dietary pattern and depressive symptoms in middle age. Br J Psychiatry 195(5):408-413.

Alwerdt J, Small BJ. 2017. Fecal incontinence as a moderator between dietary intake and depressive symptoms among a sample of older adults obtained from the National Health and Nutrition Examination Survey (NHANES). Aging Ment Health:1-11.

Assmann KE, Lassale C, Andreeva VA, Jeandel C, Hercberg S, Galan P, Kesse-Guyot E. 2015. A Healthy Dietary Pattern at Midlife, Combined with a Regulated Energy Intake, Is Related to Increased Odds for Healthy Aging. Journal of Nutrition 145(9):2139-2145.

Baskin R, Hill B, Jacka FN, O'Neil A, Skouteris H. 2017. Antenatal dietary patterns and depressive symptoms during pregnancy and early post-partum. Matern Child Nutr 13(1):01.

Bloom I, Edwards M, Jameson KA, Syddall HE, Dennison E, Gale CR, Baird J, Cooper C, Aihie Sayer A, Robinson S. 2017. Influences on diet quality in older age: the importance of social factors. Age Ageing 46(2):277-283.

Buys N, Sun J. 2013. Relationship between obesity and depression in older Australian adults and examination of dietary patterns as influencing factors. International Journal of Mental Health Promotion 15(5):263-274.

Chan R, Chan D, Woo J. 2014. A prospective cohort study to examine the association between dietary patterns and depressive symptoms in older Chinese people in Hong Kong. PLoS ONE 9(8):e105760.

Chatzi L, Melaki V, Sarri K, Apostolaki I, Roumeliotaki T, Georgiou V, Vassilaki M, Koutis A, Bitsios P, Kogevinas M. 2011. Dietary patterns during pregnancy and the risk of postpartum depression: the mother-child 'Rhea' cohort in Crete, Greece. Public Health Nutr 14(9):1663-1670.

Chocano-Bedoya PO, O'Reilly EJ, Lucas M, Mirzaei F, Okereke OI, Fung TT, Hu FB, Ascherio A. 2013. Prospective study on long-term dietary patterns and incident depression in middle-aged and older women. American Journal of Clinical Nutrition 98(3):813-820.

Denoth F, Scalese M, Siciliano V, Di Renzo L, De Lorenzo A, Molinaro S. 2016. Clustering eating habits: frequent consumption of different dietary patterns among the Italian general population in the association with obesity, physical activity, sociocultural characteristics and psychological factors. Eat Weight Disord 21(2):257-268.

Dipnall JF, Pasco JA, Meyer D, Berk M, Williams LJ, Dodd S, Jacka FN. 2015. The association between dietary patterns, diabetes and depression. J Affect Disord 174:215-224.

Engeset D, Alsaker E, Ciampi A, Lund E. 2005. Dietary patterns and lifestyle factors in the Norwegian EPIC cohort: the Norwegian Women and Cancer (NOWAC) study. European Journal of Clinical Nutrition 59(5):675-684.

Ferrand C, Feart C, Martinent G, Albinet C, Andre N, Audiffren M. 2017. Dietary patterns in French home-living older adults: Results from the PRAUSE study. Arch Gerontol Geriatr 70:180-185.

Gougeon L, Payette H, Morais J, Gaudreau P, Shatenstein B, Gray-Donald K. 2015. Dietary patterns and incidence of depression in a cohort of community-dwelling older Canadians. J Nutr Health Aging 19(4):431-436.

Gregorio MJ, Rodrigues AM, Eusebio M, Sousa RD, Dias S, Andre B, Gronning K, Coelho PS, Mendes JM, Graca P and others. 2017. Dietary Patterns Characterized by High Meat Consumption Are Associated with Other Unhealthy Life Styles and Depression Symptoms. Front 4:25.

Hosseinzadeh M, Vafa M, Esmaillzadeh A, Feizi A, Majdzadeh R, Afshar H, Keshteli AH, Adibi P. 2016. Empirically derived dietary patterns in relation to psychological disorders. Public Health Nutr 19(2):204-217.

Jaalouk D, Matar Boumosleh J, Helou L, Abou Jaoude M. 2018. Dietary patterns, their covariates, and associations with severity of depressive symptoms among university students in Lebanon: a cross-sectional study. European Journal of Nutrition 19:19.

Jacka FN, Cherbuin N, Anstey KJ, Butterworth P. 2014. Dietary patterns and depressive symptoms over time: examining the relationships with socioeconomic position, health behaviours and cardiovascular risk. PLoS ONE 9(1):e87657.

Khosravi M, Sotoudeh G, Majdzadeh R, Nejati S, Darabi S, Raisi F, Esmaillzadeh A, Sorayani M. 2015. Healthy and Unhealthy Dietary Patterns Are Related to Depression: A Case-Control Study. Psychiatry Investig 12(4):434-442.

Khosravi M, Sotoudeh G, Raisi F, Majdzadeh R, Foroughifar T. 2014. Comparing dietary patterns of depressed patients versus healthy people in a case control protocol. BMJ Open 4(2):e003843.

Kim WK, Shin D, Song WO. 2016. Are Dietary Patterns Associated with Depression in U.S. Adults? J med food 19(11):1074-1084.

Le Port A, Gueguen A, Kesse-Guyot E, Melchior M, Lemogne C, Nabi H, Goldberg M, Zins M, Czernichow S. 2012. Association between dietary patterns and depressive symptoms over time: a 10-year follow-up study of the GAZEL cohort. PLoS ONE 7(12):e51593.

Liu ZM, Ho SC, Xie YJ, Chen YJ, Chen YM, Chen B, Wong SY, Chan D, Wong CK, He Q and others. 2016. Associations between dietary patterns and psychological factors: a cross-sectional study among Chinese postmenopausal women. Menopause 23(12):1294-1302.

Lucas M, Chocano-Bedoya P, Schulze MB, Mirzaei F, O'Reilly EJ, Okereke OI, Hu FB, Willett WC, Ascherio A. 2014. Inflammatory dietary pattern and risk of depression among women.[Erratum appears in Brain Behav Immun. 2015 May;46:327 Note: Shulze, Mathias B [corrected to Schulze, Matthias B]]. Brain Behav Immun 36:46-53.

Lucas M, Chocano-Bedoya P, Shulze MB, Mirzaei F, O'Reilly EJ, Okereke OI, Hu FB, Willett WC, Ascherio A. 2015. "Inflammatory dietary pattern and risk of depression among women": Erratum. Brain, Behavior, and Immunity 46:327.

Miki T, Eguchi M, Akter S, Kochi T, Kuwahara K, Kashino I, Hu H, Kabe I, Kawakami N, Nanri A and others. 2018. Longitudinal adherence to a dietary pattern and risk of depressive symptoms: the Furukawa Nutrition and Health Study. Nutrition 48:48-54.

Miki T, Kochi T, Kuwahara K, Eguchi M, Kurotani K, Tsuruoka H, Ito R, Kabe I, Kawakami N, Mizoue T and others. 2015. Dietary patterns derived by reduced rank regression (RRR) and depressive symptoms in Japanese employees: The Furukawa nutrition and health study. Psychiatry Res 229(1-2):214-219.

Miyake Y, Tanaka K, Okubo H, Sasaki S, Furukawa S, Arakawa M. 2018. Dietary patterns and depressive symptoms during pregnancy in Japan: Baseline data from the Kyushu Okinawa Maternal and Child Health Study. J Affect Disord 225:552-558.

Nanri A, Kimura Y, Matsushita Y, Ohta M, Sato M, Mishima N, Sasaki S, Mizoue T. 2010. Dietary patterns and depressive symptoms among Japanese men and women. European Journal of Clinical Nutrition 64(8):832-839.

Nanri A, Mizoue T, Poudel-Tandukar K, Noda M, Kato M, Kurotani K, Goto A, Oba S, Inoue M, Tsugane S and others. 2013. Dietary patterns and suicide in Japanese adults: the Japan Public Health Center-based Prospective Study. Br J Psychiatry 203(6):422-427.

Noguchi R, Hiraoka M, Watanabe Y, Kagawa Y. 2013. Relationship between dietary patterns and depressive symptoms: difference by gender, and unipolar and bipolar depression. J Nutr Sci Vitaminol (Tokyo) 59(2):115-122.

Northstone K, Joinson C, Emmett P. 2018. Dietary patterns and depressive symptoms in a UK cohort of men and women: a longitudinal study. Public Health Nutr 21(5):831-837.

Oddy WH, Allen KL, Trapp GSA, Ambrosini GL, Black LJ, Huang RC, Rzehak P, Runions KC, Pan F, Beilin LJ and others. 2018. Dietary patterns, body mass index and inflammation: Pathways to depression and mental health problems in adolescents. Brain Behav Immun 69:428-439.

Oddy WH, Robinson M, Ambrosini GL, O'Sullivan TA, de Klerk NH, Beilin LJ, Silburn SR, Zubrick SR, Stanley FJ. 2009. The association between dietary patterns and mental health in early adolescence. Prev Med 49(1):39-44.

Okubo H, Miyake Y, Sasaki S, Tanaka K, Murakami K, Hirota Y, Osaka M, Child Health Study G. 2011. Dietary patterns during pregnancy and the risk of postpartum depression in Japan: the Osaka Maternal and Child Health Study. Br J Nutr 105(8):1251-1257.

Paskulin JTA, Drehmer M, Olinto MT, Hoffmann JF, Pinheiro AP, Schmidt MI, Nunes MA. 2017. Association between dietary patterns and mental disorders in pregnant women in Southern Brazil. Rev Bras Psiquiatr 39(3):208-215.

Rienks J, Dobson AJ, Mishra GD. 2013. Mediterranean dietary pattern and prevalence and incidence of depressive symptoms in mid-aged women: results from a large community-based prospective study. European Journal of Clinical Nutrition 67(1):75-82.

Ruusunen A, Lehto SM, Mursu J, Tolmunen T, Tuomainen TP, Kauhanen J, Voutilainen S. 2014. Dietary patterns are associated with the prevalence of elevated depressive symptoms and the risk of getting a hospital discharge diagnosis of depression in middle-aged or older Finnish men. J Affect Disord 159:1-6.

Samieri C, Jutand MA, Feart C, Capuron L, Letenneur L, Barberger-Gateau P. 2008. Dietary patterns derived by hybrid clustering method in older people: association with cognition, mood, and self-rated health. Journal of the American Dietetic Association 108(9):1461-1471.

Sugawara N, Yasui-Furukori N, Tsuchimine S, Kaneda A, Tsuruga K, Iwane K, Okubo N, Takahashi I, Kaneko S. 2012. No association between dietary patterns and depressive symptoms among a community-dwelling population in Japan. Ann Gen Psychiatry 11(1):24.

Suzuki T, Miyaki K, Tsutsumi A, Hashimoto H, Kawakami N, Takahashi M, Shimazu A, Inoue A, Kurioka S, Kakehashi M and others. 2013. Japanese dietary pattern consistently relates to low depressive symptoms and it is modified by job strain and worksite supports. J Affect Disord 150(2):490-498.

Teo C, Chia AR, Colega MT, Chen LW, Fok D, Pang WW, Godfrey KM, Tan KH, Yap F, Shek LP and others. 2018. Prospective Associations of Maternal Dietary Patterns and Postpartum Mental Health in a Multi-Ethnic Asian Cohort: The Growing up in Singapore towards Healthy Outcomes (GUSTO) Study. Nutrients 10(3):02.

Toyomaki A, Koga M, Okada E, Nakai Y, Miyazaki A, Tamakoshi A, Kiso Y, Kusumi I. 2017. The relationship between a low grain intake dietary pattern and impulsive behaviors in middle-aged Japanese people. PLoS ONE 12(7):e0181057.

Tsai HJ. 2016. Dietary patterns and depressive symptoms in a Taiwanese population aged 53 years and over: Results from the Taiwan Longitudinal Study of Aging. Geriatr Gerontol Int 16(12):1289-1295.

Vermeulen E, Brouwer IA, Stronks K, Bandinelli S, Ferrucci L, Visser M, Nicolaou M. 2018. Inflammatory dietary patterns and depressive symptoms in Italian older adults. Brain Behav Immun 67:290-298.

Vermeulen E, Stronks K, Snijder MB, Schene AH, Lok A, de Vries JH, Visser M, Brouwer IA, Nicolaou M. 2017a. A combined high-sugar and high-saturated-fat dietary pattern is associated with more depressive symptoms in a multi-ethnic population: the HELIUS (Healthy Life in an Urban Setting) study. Public Health Nutr 20(13):2374-2382.

Vermeulen E, Stronks K, Visser M, Brouwer IA, Schene AH, Mocking RJ, Colpo M, Bandinelli S, Ferrucci L, Nicolaou M. 2016. The association between dietary patterns derived by reduced rank regression and depressive symptoms over time: the Invecchiare in Chianti (InCHIANTI) study. Br J Nutr 115(12):2145-2153.

Vermeulen E, Stronks K, Visser M, Brouwer IA, Snijder MB, Mocking RJT, Derks EM, Schene AH, Nicolaou M. 2017b. Dietary pattern derived by reduced rank regression and depressive symptoms in a multi-ethnic population: the HELIUS study. European Journal of Clinical Nutrition 71(8):987-994.

Vilela AA, Farias DR, Eshriqui I, Vaz Jdos S, Franco-Sena AB, Castro MB, Olinto MT, Machado SP, Moura da Silva AA, Kac G. 2014. Prepregnancy healthy dietary pattern is inversely associated with depressive symptoms among pregnant Brazilian women. Journal of Nutrition 144(10):1612-1618.

Wang CJ, Yang TF, Wang GS, Zhao YY, Yang LJ, Bi BN. 2017. Association between dietary patterns and depressive symptoms among middle-aged adults in China in 2016-2017. Psychiatry Res 260:123-129.

Weng TT, Hao JH, Qian QW, Cao H, Fu JL, Sun Y, Huang L, Tao FB. 2012. Is there any relationship between dietary patterns and depression and anxiety in Chinese adolescents? Public Health Nutr 15(4):673-682.

Xia Y, Wang N, Yu B, Zhang Q, Liu L, Meng G, Wu H, Du H, Shi H, Guo X and others. 2017. Dietary patterns are associated with depressive symptoms among Chinese adults: a case-control study with propensity score matching. European Journal of Nutrition 56(8):2577-2587.

Yazdi M, Roohafza H, Feizi A, Rabiei K, Sarafzadegan N. 2018. The influence of dietary patterns and stressful life events on psychological problems in a large sample of Iranian industrial employees: Structural equations modeling approach. J Affect Disord 236:140-148.

**Reason**: Vegetarian diet

Beezhold B, Radnitz C, Rinne A, DiMatteo J. 2015. Vegans report less stress and anxiety than omnivores. Nutr Neurosci 18(7):289-296.

Beezhold BL, Johnston CS, Daigle DR. 2010. Vegetarian diets are associated with healthy mood states: a cross-sectional study in seventh day adventist adults. Nutr J 9:26.

Hibbeln JR, Northstone K, Evans J, Golding J. 2018. Vegetarian diets and depressive symptoms among men. J Affect Disord 225:13-17.

Kahleova H, Hrachovinova T, Hill M, Pelikanova T. 2013. Vegetarian diet in type 2 diabetes--improvement in quality of life, mood and eating behaviour. Diabetic Medicine 30(1):127-129.

Velten J, Bieda A, Scholten S, Wannemuller A, Margraf J. 2018. Lifestyle choices and mental health: a longitudinal survey with German and Chinese students. BMC Public Health 18(1):632.

**Reason**: Glycaemic index

Aparicio A, Robles F, Lopez-Sobaler AM, Ortega RM. 2013. Dietary glycaemic load and odds of depression in a group of institutionalized elderly people without antidepressant treatment. European Journal of Nutrition 52(3):1059-1066.

Gangwisch JE, Hale L, Garcia L, Malaspina D, Opler MG, Payne ME, Rossom RC, Lane D. 2015. High glycemic index diet as a risk factor for depression: analyses from the Women's Health Initiative. American Journal of Clinical Nutrition 102(2):454-463.

Gopinath B, Flood VM, Burlutksy G, Louie JC, Mitchell P. 2016. Association between carbohydrate nutrition and prevalence of depressive symptoms in older adults. Br J Nutr 116(12):2109-2114.

Haghighatdoost F, Azadbakht L, Keshteli AH, Feinle-Bisset C, Daghaghzadeh H, Afshar H, Feizi A, Esmaillzadeh A, Adibi P. 2016. Glycemic index, glycemic load, and common psychological disorders. American Journal of Clinical Nutrition 103(1):201-209.

Minobe N, Murakami K, Kobayashi S, Suga H, Sasaki S, Three-generation Study of Women on D, Health Study G. 2017. Higher dietary glycemic index, but not glycemic load, is associated with a lower prevalence of depressive symptoms in a cross-sectional study of young and middle-aged Japanese women. European Journal of Nutrition 20:20.

Murakami K, Miyake Y, Sasaki S, Tanaka K, Yokoyama T, Ohya Y, Fukushima W, Kiyohara C, Hirota Y, Osaka M and others. 2008. Dietary glycemic index and load and the risk of postpartum depression in Japan: the Osaka Maternal and Child Health Study. J Affect Disord 110(1-2):174-179.

**Reason**: Pregnancy

Chang MW, Brown R, Nitzke S. 2016. Fast Food Intake in Relation to Employment Status, Stress, Depression, and Dietary Behaviors in Low-Income Overweight and Obese Pregnant Women. Matern Child Health J 20(7):1506-1517.

Chang MW, Brown R, Nitzke S, Smith B, Eghtedary K. 2015. Stress, sleep, depression and dietary intakes among low-income overweight and obese pregnant women. Matern Child Health J 19(5):1047-1059.

Chatzi L, Melaki V, Sarri K, Apostolaki I, Roumeliotaki T, Georgiou V, Vassilaki M, Koutis A, Bitsios P, Kogevinas M. 2011. Dietary patterns during pregnancy and the risk of postpartum depression: the mother-child 'Rhea' cohort in Crete, Greece. Public Health Nutr 14(9):1663-1670.

Emmett PM, Jones LR, Golding J. 2015. Pregnancy diet and associated outcomes in the Avon Longitudinal Study of Parents and Children. Nutr Rev 73 Suppl 3:154-174.

Jans G, Matthys C, Bogaerts A, Ameye L, Delaere F, Roelens K, Loccufier A, Logghe H, De Becker B, Verhaeghe J and others. 2018. Depression and Anxiety: Lack of Associations with an Inadequate Diet in a Sample of Pregnant Women with a History of Bariatric Surgery-a Multicenter Prospective Controlled Cohort Study. Obesity Surgery 28(6):1629-1635.

Miyake Y, Tanaka K, Okubo H, Sasaki S, Furukawa S, Arakawa M. 2018. Dietary patterns and depressive symptoms during pregnancy in Japan: Baseline data from the Kyushu Okinawa Maternal and Child Health Study. J Affect Disord 225:552-558.

Okubo H, Miyake Y, Sasaki S, Tanaka K, Murakami K, Hirota Y, Osaka M, Child Health Study G. 2011. Dietary patterns during pregnancy and the risk of postpartum depression in Japan: the Osaka Maternal and Child Health Study. Br J Nutr 105(8):1251-1257.

Paskulin JTA, Drehmer M, Olinto MT, Hoffmann JF, Pinheiro AP, Schmidt MI, Nunes MA. 2017. Association between dietary patterns and mental disorders in pregnant women in Southern Brazil. Rev Bras Psiquiatr 39(3):208-215.

Vilela AA, Farias DR, Eshriqui I, Vaz Jdos S, Franco-Sena AB, Castro MB, Olinto MT, Machado SP, Moura da Silva AA, Kac G. 2014. Prepregnancy healthy dietary pattern is inversely associated with depressive symptoms among pregnant Brazilian women. Journal of Nutrition 144(10):1612-1618.

**Reason**: Intervention studies

Agarwal U, Mishra S, Xu J, Levin S, Gonzales J, Barnard ND. 2015. A multicenter randomized controlled trial of a nutrition intervention program in a multiethnic adult population in the corporate setting reduces depression and anxiety and improves quality of life: the GEICO study. Am J Health Promot 29(4):245-254.

Ali S, de Araujo Pio CS, Chaves GSS, Britto R, Cribbie R, Grace SL. 2018. Psychosocial well-being over the two years following cardiac rehabilitation initiation & association with heart-health behaviors. Gen Hosp Psychiatry 52:48-57.

Assaf AR, Beresford SA, Risica PM, Aragaki A, Brunner RL, Bowen DJ, Naughton M, Rosal MC, Snetselaar L, Wenger N. 2016. Low-Fat Dietary Pattern Intervention and Health-Related Quality of Life: The Women's Health Initiative Randomized Controlled Dietary Modification Trial. J Acad Nutr Diet 116(2):259-271.

Baker AL, Richmond R, Kay-Lambkin FJ, Filia SL, Castle D, Williams JM, Lewin TJ, Clark V, Callister R, Palazzi K. 2018. Randomised controlled trial of a healthy lifestyle intervention among smokers with psychotic disorders: Outcomes to 36months. Aust N Z J Psychiatry 52(3):239-252.

Brinkworth GD, Buckley JD, Noakes M, Clifton PM, Wilson CJ. 2009. Long-term effects of a very low-carbohydrate diet and a low-fat diet on mood and cognitive function. Archives of Internal Medicine 169(20):1873-1880.

Brinkworth GD, Luscombe-Marsh ND, Thompson CH, Noakes M, Buckley JD, Wittert G, Wilson CJ. 2016. Long-term effects of very low-carbohydrate and high-carbohydrate weight-loss diets on psychological health in obese adults with type 2 diabetes: randomized controlled trial. J Intern Med 280(4):388-397.

Chatterton ML, Mihalopoulos C, O'Neil A, Itsiopoulos C, Opie R, Castle D, Dash S, Brazionis L, Berk M, Jacka F. 2018. Economic evaluation of a dietary intervention for adults with major depression (the "SMILES" trial). BMC Public Health 18(1):599.

Chlebowy DO, Batscha C, Kubiak N, Crawford T. 2018. Relationships of Depression, Anxiety, and Stress with Adherence to Self-Management Behaviors and Diabetes Measures in African American Adults with Type 2 Diabetes. J Racial Ethn Health Disparities 29:29.

Ciarambino T, Castellino P, Paolisso G, Coppola L, Ferrara N, Signoriello G, Giordano M. 2012a. Long term effects of low protein diet on depressive symptoms and quality of life in elderly Type 2 diabetic patients. Clin Nephrol 78(2):122-128.

Ciarambino T, Ferrara N, Castellino P, Paolisso G, Coppola L, Giordano M. 2012b. Effects of six days a week low protein dietary intervention on depressive symptoms in elderly diabetic subjects. Giornale di Gerontologia 60(1):8-13.

Garcia-Toro M, Vicens-Pons E, Gili M, Roca M, Serrano-Ripoll MJ, Vives M, Leiva A, Yanez AM, Bennasar-Veny M, Olivan-Blazquez B. 2016. Obesity, metabolic syndrome and Mediterranean diet: Impact on depression outcome. J Affect Disord 194:105-108.

Knight A, Bryan J, Wilson C, Hodgson J, Murphy K. 2015. A randomised controlled intervention trial evaluating the efficacy of a Mediterranean dietary pattern on cognitive function and psychological wellbeing in healthy older adults: the MedLey study. BMC geriatr 15:55.

Martinez-Gonzalez MA, Sanchez-Villegas A. 2016. Food patterns and the prevention of depression. Proc Nutr Soc 75(2):139-146.

O'Neil A, Berk M, Itsiopoulos C, Castle D, Opie R, Pizzinga J, Brazionis L, Hodge A, Mihalopoulos C, Chatterton ML and others. 2013. A randomised, controlled trial of a dietary intervention for adults with major depression (the "SMILES" trial): study protocol. BMC Psychiatry 13:114.

Opie RS, O'Neil A, Jacka FN, Pizzinga J, Itsiopoulos C. 2017. A modified Mediterranean dietary intervention for adults with major depression: Dietary protocol and feasibility data from the SMILES trial. Nutr Neurosci:1-15.

Parletta N, Zarnowiecki D, Cho J, Wilson A, Bogomolova S, Villani A, Itsiopoulos C, Niyonsenga T, Blunden S, Meyer B and others. 2017. A Mediterranean-style dietary intervention supplemented with fish oil improves diet quality and mental health in people with depression: A randomized controlled trial (HELFIMED). Nutr Neurosci:1-14.

Sanchez-Villegas A, Martinez-Gonzalez MA, Estruch R, Salas-Salvado J, Corella D, Covas MI, Aros F, Romaguera D, Gomez-Gracia E, Lapetra J and others. 2013. Mediterranean dietary pattern and depression: the PREDIMED randomized trial. BMC Med 11:208.

Sorensen M, Anderssen S, Hjerman I, Holme I, Ursin H. 1999. The effect of exercise and diet on mental health and quality of life in middle-aged individuals with elevated risk factors for cardiovascular disease. J Sports Sci 17(5):369-377.

Stiekema APM, Looijmans A, van der Meer L, Bruggeman R, Schoevers RA, Corpeleijn E, Jorg F. 2018. Effects of a lifestyle intervention on psychosocial well-being of severe mentally ill residential patients: ELIPS, a cluster randomized controlled pragmatic trial. Schizophr Res 01:01.

Torres SJ, Nowson CA. 2012. A moderate-sodium DASH-type diet improves mood in postmenopausal women. Nutrition 28(9):896-900.

**Reason**: Systematic reviews

Baskin R, Hill B, Jacka FN, O'Neil A, Skouteris H. 2015. The association between diet quality and mental health during the perinatal period. A systematic review. Appetite 91:41-47.

Cairns KE, Yap MB, Pilkington PD, Jorm AF. 2014. Risk and protective factors for depression that adolescents can modify: a systematic review and meta-analysis of longitudinal studies. J Affect Disord 169:61-75.

Feng Z, Lugtenberg M, Franse C, Fang X, Hu S, Jin C, Raat H. 2017. Risk factors and protective factors associated with incident or increase of frailty among community-dwelling older adults: A systematic review of longitudinal studies. PLoS ONE 12(6):e0178383.

Jacka FN, Berk M. 2013. Depression, diet and exercise. Med J Aust 199(6 Suppl):S21-23.

Khalid S, Williams CM, Reynolds SA. 2016. Is there an association between diet and depression in children and adolescents? A systematic review. Br J Nutr 116(12):2097-2108.

Lai JS, Hiles S, Bisquera A, Hure AJ, McEvoy M, Attia J. 2014. A systematic review and meta-analysis of dietary patterns and depression in community-dwelling adults. American Journal of Clinical Nutrition 99(1):181-197.

Li Y, Lv MR, Wei YJ, Sun L, Zhang JX, Zhang HG, Li B. 2017. Dietary patterns and depression risk: A meta-analysis. Psychiatry Res 253:373-382.

Lopresti AL, Jacka FN. 2015. Diet and Bipolar Disorder: A Review of Its Relationship and Potential Therapeutic Mechanisms of Action. J Altern Complement Med 21(12):733-739.

Molendijk M, Molero P, Ortuno Sanchez-Pedreno F, Van der Does W, Angel Martinez-Gonzalez M. 2018. Diet quality and depression risk: A systematic review and dose-response meta-analysis of prospective studies. J Affect Disord 226:346-354.

Opie RS, Itsiopoulos C, Parletta N, Sanchez-Villegas A, Akbaraly TN, Ruusunen A, Jacka FN. 2017. Dietary recommendations for the prevention of depression. Nutr Neurosci 20(3):161-171.

Opie RS, O'Neil A, Itsiopoulos C, Jacka FN. 2015. The impact of whole-of-diet interventions on depression and anxiety: a systematic review of randomised controlled trials. Public Health Nutr 18(11):2074-2093.

Quirk SE, Williams LJ, O'Neil A, Pasco JA, Jacka FN, Housden S, Berk M, Brennan SL. 2013. The association between diet quality, dietary patterns and depression in adults: a systematic review. BMC Psychiatry 13:175.

Rahe C, Berger K. 2016. Nutrition and depression: Current evidence on the association of dietary patterns with depression and its subtypes. Cardiovascular diseases and depression: Treatment and prevention in psychocardiology. Cham, Switzerland: Springer International Publishing; Switzerland. p 279-304.

Rahe C, Unrath M, Berger K. 2014. Dietary patterns and the risk of depression in adults: a systematic review of observational studies. European Journal of Nutrition 53(4):997-1013.

Rahimlou M, Morshedzadeh N, Karimi S, Jafarirad S. 2018. Association between dietary glycemic index and glycemic load with depression: a systematic review. European Journal of Nutrition 09:09.

Sanhueza C, Ryan L, Foxcroft DR. 2013. Diet and the risk of unipolar depression in adults: systematic review of cohort studies. J Hum Nutr Diet 26(1):56-70.
